# TropMol-Caipora: A Cloud-Based Web Tool to Predict Cruzain Inhibitors by Machine Learning

**DOI:** 10.1021/acsomega.5c08795

**Published:** 2026-01-08

**Authors:** Thiago H. Doring

**Affiliations:** Department of Exact Sciences and Education (CEE), School of Technology, Exact Sciences and Education (CTE), Federal University of Santa Catarina (UFSC), Blumenau 89036-256, SC, Brazil

## Abstract

Chagas disease (CD) affects approximately 8 million people and is classified as a high-priority neglected tropical disease by the WHO research and development actions. One promising avenue for drug development for CD is the inhibition of cruzain, a crucial cysteine protease of *T. cruzi* and one of the most extensively studied therapeutic targets. This study aims to construct a generic molecular screening model for public, online, and free use, based on pIC_50_ cruzain predictions using a Random Forest model. For this, a data set with approximately 8 thousand compounds and 168 classes of descriptors was used, resulting in more than a million calculated descriptors. The model achieved *R* ^2^ = 0.91 (RMSE = 0.33) for the training set and *R* ^2^ = 0.72 (RMSE = 0.55) for the test set. In 5-fold cross-validation, performance remained consistent (*R* ^2^ = 0.72 ± 0.01; RMSE = 0.57 ± 0.01). Some relevant insights were also observed. 1 - Aromaticity was shown to be a key factor in inhibitory activity. Compounds with nitrogenous aromatic rings are more likely to be more effective inhibitors. Aromatics in general also present correlation and structural relevance for an effective inhibitor. 2 - Halogenation may favor activity. The positive correlation may suggest that the introduction of halogen atoms may improve the activity of the compounds. 3 - Bicyclic or very rigid structures may decrease the inhibition efficiency of the tested candidates. 4 - Molecular accessibility and charge influence activity. Available in: https://colab.research.google.com/drive/1hotsXPddbJ6E0_hysLT9AqsXL-74Na-z?usp=sharing.

## Graphical abstract

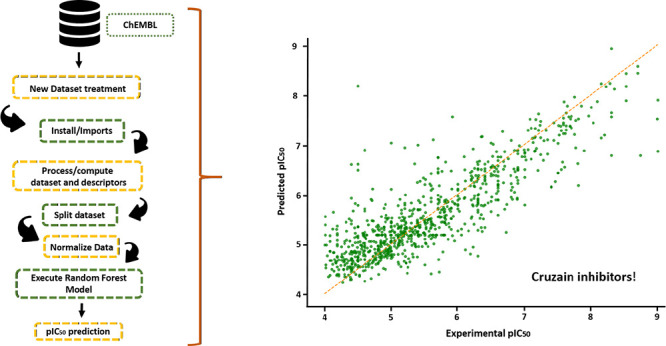

## Introduction

Chagas disease (CD) affects approximately 8 million people and is classified as a high-priority neglected tropical disease (NTD) by the World Health Organization (WHO) research and development actions.^1^ Caused by the protozoan *Trypanosoma cruzi*, the disease predominantly affects socially vulnerable populations in Latin America. In addition to being endemic in several Latin American countries, the disease has spread to North America and, to a lesser extent, to some countries in Europe, Asia, and Oceania, driven by migratory movements.^1^

The parasite is transmitted through the feces of insects known as “kissing bugs”, such as *Triatoma infestans*, which feed on the host’s blood. After a short acute phase, the patient may remain asymptomatic for several decades. The parasite can contaminate the site of the bite and, after an acute phase, the disease is often diagnosed only in its chronic phase. Serious problems such as cardiomegaly, megaesophagus or megacolon may appear.^2^ These conditions can lead to a significant loss of quality of life and even early death.^3^

Although the disease was identified and characterized more than a century ago by the Brazilian scientist Carlos Chagas,^4^ the available therapeutic options are quite limited. The existing drugs, benznidazole and nifurtimox, were developed in the 1970s and generally present serious problems, such as low efficacy in the chronic phase and high toxicity.^5^ The need for new drugs is urgent due to the reduced efficacy in the chronic phase and the lack of investment by the global pharmaceutical industry.^6,7^

One promising avenue for drug development is the inhibition of cruzain, a crucial cysteine protease of *T. cruzi* and one of the most extensively studied therapeutic targets. It plays a vital role in the parasite’s survival by facilitating key processes such as host cell invasion and proliferation.^8^ The protein’s active site is divided into seven subsites that interact with substrates, labeled S1 to S4 on the acyl side and S1′ to S3′ on the amino side. However, the subsites S1, S2, S3, and S1′ are the most relevant for inhibitor interactions, making them critical for drug development studies.^9^ Inhibiting cruzain significantly reduces the parasite load, as low enzyme concentrations impair the protozoan’s ability to invade host cells. Consequently, cruzain is a promising target for the development of new treatments for Chagas disease. Virtual screening techniques are particularly useful in the early stages of drug discovery, allowing for the theoretical identification of optimal molecular structures and functional groups that interact effectively with the enzyme’s active site.^10^

Computer-aided drug design (CADD) methods have become increasingly valuable for screening extensive compound databases to identify potential inhibitors.^11,12^ One CADD strategy that has significantly advanced drug discovery is the application of machine learning (ML) algorithms. Common ML techniques in CADD range from traditional models like logistic regression, random forest (RF), and support vector machine (SVM) to more advanced approaches, including extreme gradient boosting (XGBoost) and deep neural networks (DNN).

Previous studies have explored targeted data sets to predict properties closely correlated with IC_50_ against cruzain.^9,13,14^ In this context, general drug design models can be valuable tools for research groups focused on chemical and biochemical synthesis, aiding in virtual screening (VS).^15–17^ This study aims to develop a general prediction model for molecular screening, freely accessible online, to estimate pIC_50_ against cruzain using the Google Colab framework and a random forest (RF) model. The data set comprises approximately 8000 compounds and 168 descriptor classes, leading to the calculation of over a million descriptors. Additionally, this work highlights key insights into descriptor relevance, which are essential for understanding cruzain inhibitors.

## Methodology

To predict pIC_50_ values, the data set was initially collected from ChEMBL.^18^ Duplicate entries were removed. Descriptor values (totaling approximately 1.5 million entries) were then computed.

Predictors were built using the RDKit Chem Descriptors package,^19^ which initially generated 208 descriptors. After eliminating those with high correlations (exceeding 95%), 168 descriptors remained for constructing the model. The 95% threshold for correlation-based descriptor removal was chosen to balance redundancy reduction with information retention.^20,21^ The descriptor calculations were checked to ensure there were no non-numeric, missing, or infinite values. All values were standardized using scikit-learn’s StandardScaler module.^22^ Details on the tuning parameters are provided in the Supporting Information. The model workflow is illustrated in Figure 1.

**Figure 1:**
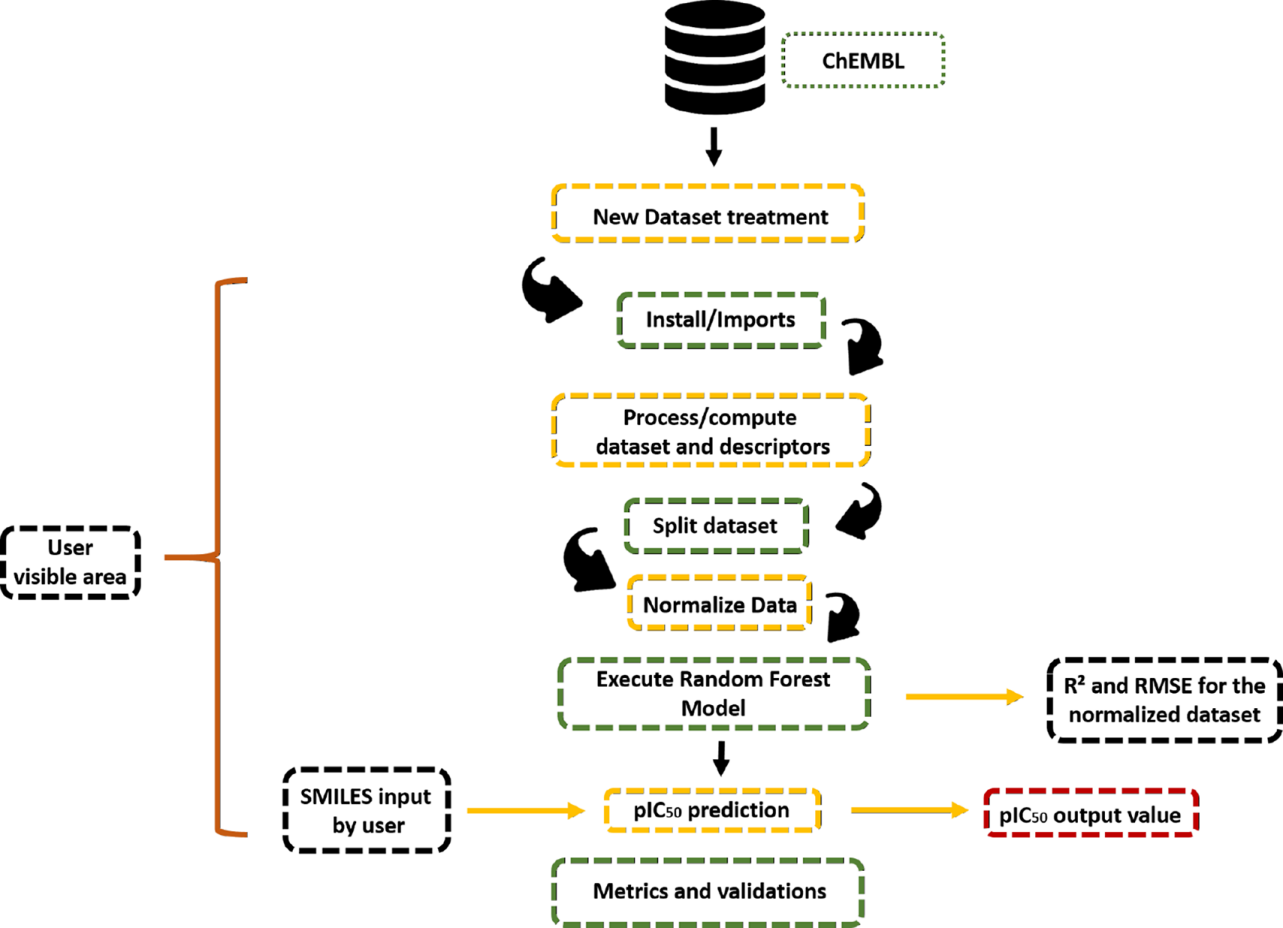
Workflow of the RF model showed by Jupyter notebook.

## Results and Discussion

The data set consists of 8829 compounds, where 1,483,104 descriptors were computed. The pIC_50_ values are distributed with a lower quartile of 4.75 and an upper quartile of 6.22. In this collection, pIC_50_ values range from a minimum of 4.00 to a maximum of 10.00. The average value found was 5.57, and a standard deviation of 1.42. It is evident that compounds exhibiting either low to moderate inhibitory activity (pIC_50_ below 4.00) are underrepresented in the literature (Table 1).

**Table 1:** pIC_50_ Variability^a^

count	8829
mean	5.57
Std	1.07
Min	4.00
25%	4.75
50%	5.31
75%	6.22
Max	10.00

a Count = Total number of compounds in the dataset; Mean = Average pIC_50_ value; Std = Dispersion of pIC_50_ values; Min = The lowest pIC_50_ value found in the dataset; 25% = Lower quartile; 50% = Median; 75% = Upper quartile; Max = The highest pIC_50_ value found in the dataset.

It has been reported that cruzain activity values are influenced by assay conditions, including the presence of detergent.^23^ However, because heterogeneous experimental sources are integrated into the database, specific details (e.g., detergent use) are not always consistently annotated. Instead, the large data set size and the external validation were used to mitigate potential biases and improve generalization. To study and evaluate the model, a 10% test set was randomly determined. The hyperparameters were tuned using *n_estimators*: 100; *max_depth*: 30; *min_samples_leaf*: 1; *max_features*: sqrt (*R* ^2^ = 0.72, RMSE = 0.55; Figure 2). The hyperparameters used for model training are available in Supplementary Data. A 5% data set was randomly constructed for end-user prediction.

**Figure 2:**
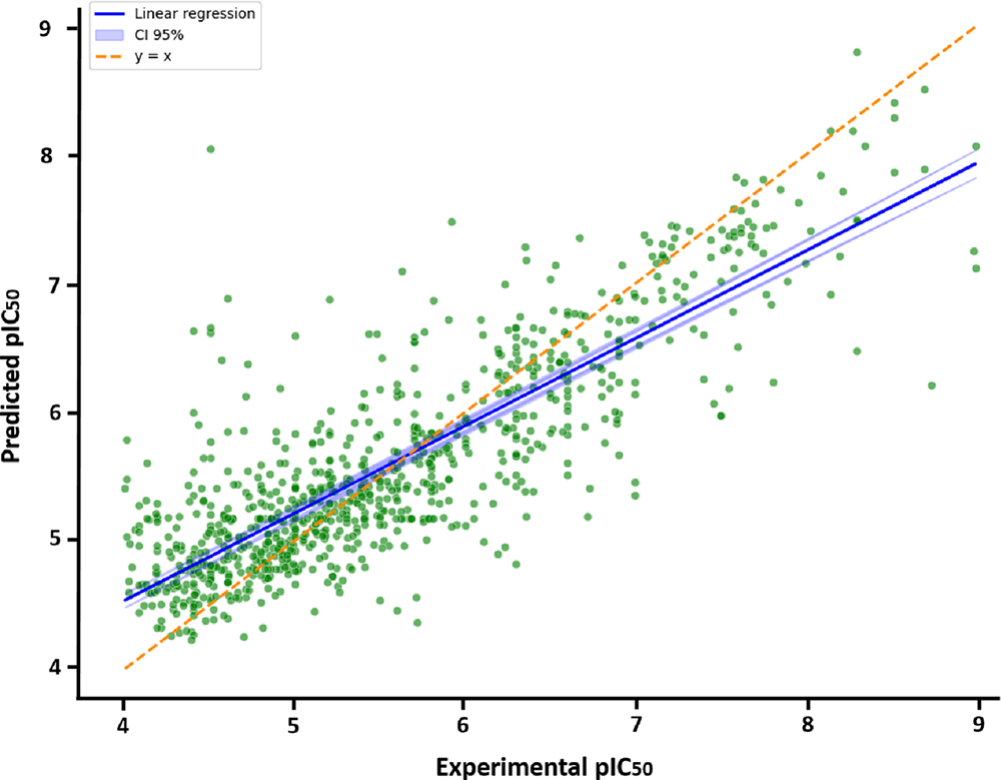
Correlation between predicted pIC_50_ and experimental pIC_50_ for the test set (test size: 10%, 883 compounds. CI = Confidence interval).

Based on the preliminary results, a 5-Fold Cross-Validation was conducted using the optimized hyperparameters. Performance metrics were gathered for both the 10% test set and the 5% test set (Table 2). The average *R* ^2^ suggests that the model accounts for approximately 72% of the variance in the 10% test set and 73% in the end-user model. The training set *R* ^2^ is moderately higher than the test set *R* ^2^, indicating acceptable generalization. Cross-validation yielded consistent results with a standard deviation of 0.009 in *R* ^2^. Y-scrambling test (100 iterations) was performed to evaluate model robustness (Supporting Information). The average *R* ^2^ of the scrambled models was −0.21 ± 0.05, and the average RMSE was 1.18 ± 0.04, in contrast with the original model (*R* ^2^ = 0.72–0.73; RMSE = 0.55). These results indicate that the model does not capture chance correlations. Together, these analyses show that the model generalizes reasonably well.

**Table 2:** 5-Fold Cross-Validation Using Normalized Dataset (5% Test and 10% Test Sets)^a^

5-fold cross-validation	5% test set	10% test set
absolute *R* ^2^ (training)	0.91	0.91
absolute *R* ^2^ (test)	0.73	0.72
absolute RMSE (training)	0.33	0.33
absolute RMSE (test)	0.55	0.55
stratified *R* ^2^ (training)	0.91	0.91
stratified *R* ^2^ (test)	0.72	0.73
stratified RMSE (training)	0.32	0.32
stratified RMSE (test)	0.56	0.54
*R* ^2^ (*x̅*)	0.72	0.71
RMSE (x̅)	0.57	0.57
MAE (*x̅*)	0.41	0.41
MPD (*x̅*)	0.08	0.08
MGD (*x̅*)	0.05	0.05
*R* ^2^ (σ)	0.01	0.009
RMSE (σ)	0.01	0.01
MAE (σ)	0.009	0.01
MPD (σ)	0.002	0.002
MGD (σ)	0.001	0.002

a MAE = Mean absolute error; RMSE = Root mean square deviation; *R* ^2^ = determination coefficient; MPD = mean percentage deviation; MGD = median geometric deviation.

Additionally, the standard deviation of *R* ^2^ values indicates consistency across different data set subsets, highlighting the model’s generalization capability. To mitigate potential bias arising from the underrepresentation of weakly active compounds, a stratified sampling approach was employed during data partitioning. The continuous pIC_50_ values were discretized into four activity bins (<5, 5–6, 6–7, >7), and stratification was applied during the train/test split to preserve the relative distribution of each activity range across both subsets (Table 2).

The Mean Absolute Error (MAE) suggests that, on average, the model’s predictions differ from the true pIC_50_ values by around 0.4 units. The Root Mean Squared Error (RMSE) is 0.57 for both test sets. The Mean Percentage Deviation (MPD) reveals that the model’s predictions, on average, deviate by 8% from the actual pIC_50_ values across both test sets. Furthermore, the Median Geometric Deviation indicates that half of the percentage deviations are below 5%, highlighting a consistent predictive performance (Figure 3).

**Figure 3:**
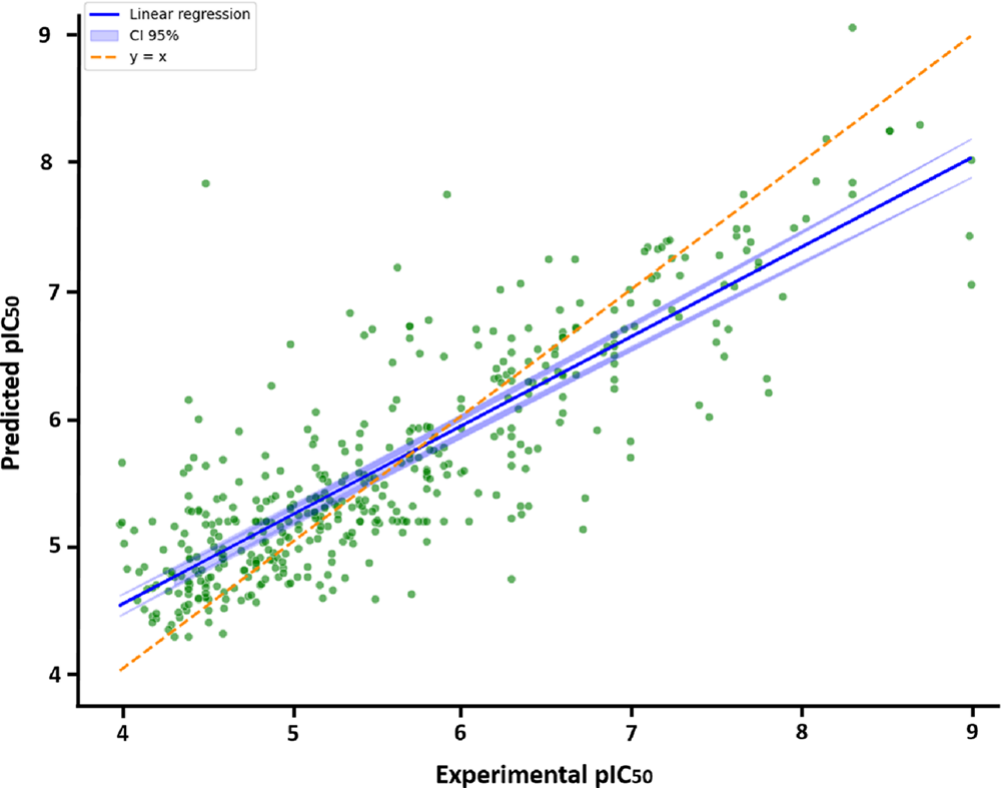
Correlation between predicted pIC_50_ and experimental pIC_50_ for the test set (test size: 5%, 442 compounds. CI = Confidence interval).

To build the model, 100 decision trees were used, with a maximum depth of 30 nodes. Each decision tree has *R* ^2^ ∼ 0.4 and RMSE ∼ 0.7, which is considerably improved when using RF, observing an increase in *R* ^2^ and a decrease in RMSE (Figure 4A). Figure 4B shows the dispersion of the individual predictions of each Decision Tree (colored dots) compared to the average prediction of RF (red dots). The dispersion of the individual points suggests that each Decision Tree has noisier predictions than the RF model. The average of the RF predictions (red dots) aligns better with the diagonal, indicating greater accuracy.

**Figure 4:**
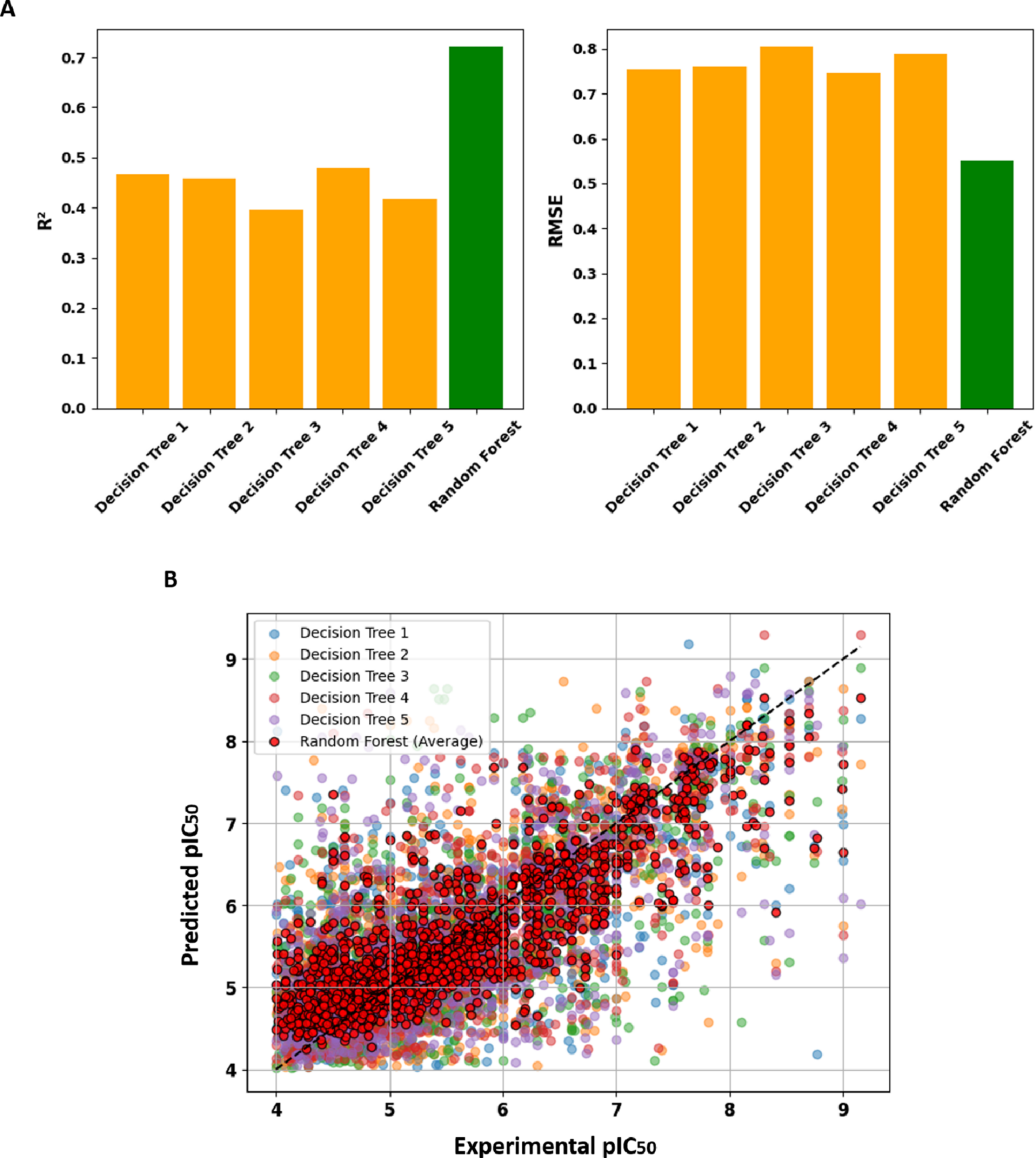
(A) Comparison of *R* ^2^ and RMSE of the first five decision trees of the model with the total RF model (*n_estimators*: 100; *max_depth*: 30; *min_samples_leaf*: 1; *max_features*: sqrt). (B) Variation of the obtained predicted pIC_50_ values between the individual decision trees and the final RF model.

### External Validation

For external validation, structures explored in three different works that are not present in the data set were used. The vinyl sulfone-derived standard K11777, present in cruzain deposited in the PDB (ID: 2OZ2, Table 3, absent in the data set), was also used.

**Table 3:** External Validation Using Normalized Dataset (5% Test Set)

ID	SMILES^a^	pIC_50_ (exp)	pIC_50_ (pred)
18 ^24^	CC(C)(CN)CNc1ccnc2cc(Cl)ccc12	4.6	5.06
19 ^24^	Clc1ccc2c(N3CCCC3)ccnc2c1	4.42	4.84
20 ^24^	CN(C)c1ccnc2cc(Cl)ccc12	4.26	4.97
21 ^24^	C#CCNCCNc1ccnc2ccCl)ccc12	4.64	4.83
20 ^25^	NC(=O)Cn1c(CCNC(=O)COc2ccccc2)nc2ccccc21	4.92	5.05
21 ^25^	O=C(COc1ccc2cc(O)ccc2c1)NCCc1nc2ccccc2[nH]1	5.47	4.94
22 ^25^	O=C(COc1ccc2ccc(O)cc2c1)NCCc1nc2ccccc2[nH]1	5.57	4.96
24 ^25^	CC(C)[C@](C)(NCc1ccc2ncccc2c1)c1cn([C@@H](CCS(C)(=O)=O)C(=O)COc2c(F)c(F)cc(F)c2F)nn1	6.25	6.68
25 ^25^	CCCC[C@@H](C(=O)COc1c(F)c(F)cc(F)c1F)n1cc([C@@](C)(NCc2ccc3ncccc3c2)C2CCCC2)nn1	7.57	6.54
8 ^26^	CN1C(=O)/C(=N\NC(N)=S)c2cc(Cl)ccc21	4.98	5.02
36 ^26^	O=C(NCO)N/*N=*C/c1ccc(NOO)o1	4.98	5.11
8 ^27^	O=C(NCc1ccco1)C1Cc2ccccc2CN1	5.52	5.06
22 ^27^	O=C(NCc1ccco1)[C@@H]1Cc2c(F)cccc2CN1	4.59	5.2
K11777 ^28^	CN1CCN(C(=O)NC(Cc2ccccc2)C(=O)NC(CCc2ccccc2)CCS(=O)(=O)c2ccccc2)CC1	5.71	5.27
Nfz-1 ^29^	O=C(N/*N=*C/c1ccc([N+](=O)[O−])s1)c1ccc(O)cc1	4.75	5.14
Nfz-4 ^29^	O=C(N/*N*=C/c1ccco1)c1ccc(O)cc1	4.16	4.88
Nfz-8 ^29^	CCc1ccc(/C=N/NC(=O)c2ccc(O)cc2)cc1	5.34	4.86

a SMILES = Simplified Molecular-Input Line-Entry System; pIC_50_ (exp) = Experimental pIC_50_; pIC_50_ (pred) = Predicted pIC_50_. Dif = Difference between experimental and predicted values.

Structures 18, 19, 20, and 21 (a) are chloroquinoline derivatives. Structures 20, 21, 22 (b) are benzimidazoles, 24 and 25 (b) are quinoline derivatives. 8 (c) is thiosemicarbazone and 36 (c) is hydrazine. 8 (d) and 22 (d) are isoquinoline derivatives. Structures Nfz-1, Nfz-4, and Nfz-8 are 4-Hydroxybenzhydrazone derivatives. The structures mentioned in the literature showed theoretical-experimental correlation *R* ^2^ = 0.60 (Figure 5).

**Figure 5:**
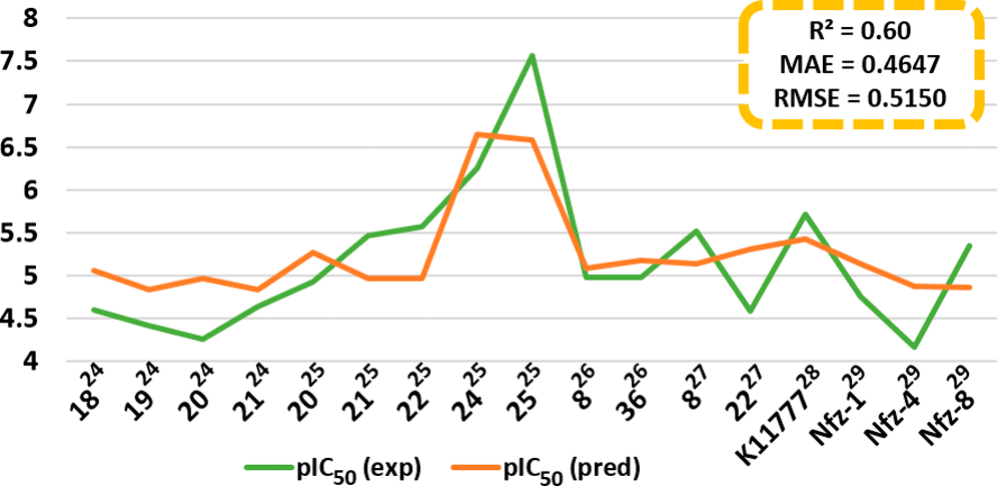
Difference between experimental pIC_50_ and predicted pIC_50_ for all compounds in the external validation set.

### Descriptors

Analysis of the descriptor correlation matrix showed that many correlations were near zero, suggesting that the descriptors generally provide complementary and distinct information (Figure 6). In contrast, groups of descriptors involving van der Waals Surface Area (VSA) tended to exhibit stronger mutual correlations, both positive and negative. Notably, the descriptors most strongly correlated with biological activity were fr_pyridine, NumAromaticHeterocycles, SMR_VSA7, PEOE_VSA8, and fr_halogen (Table 4).

**Figure 6:**
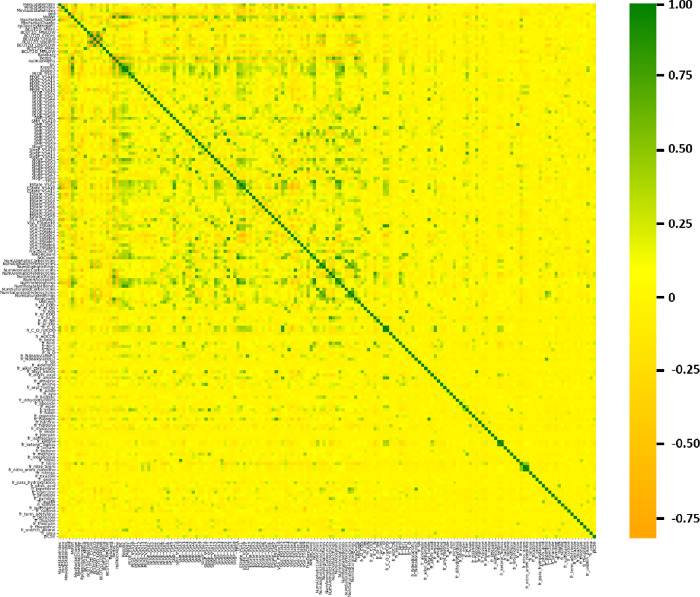
Correlation matrix between descriptors and pIC_50_.

**Table 4:** Descriptors Correlated with pIC_50_

fr_pyridine	0.33
NumAromaticHeterocycles	0.29
SMR_VSA7	0.29
PEOE_VSA8	0.27
fr_halogen	0.27
fr_ketone_Topliss	–0.17
fr_bicyclic	–0.20
MinStateIndex	–0.21

fr_pyridine descriptor indicates the presence of pyridine groups in the structure. The correlation suggests that pyridine groups contribute to cruzain inhibition. NumAromaticHeterocycles represents the number of aromatic heterocycles in the molecule. It is suggested that the inhibitory activity is greater with the presence of aromatic heterocycles in the structure, with greater specificity for pyridine groups.^19^ fr_halogen indicates the halogen moiety of the structure, which may increase the lipophilicity of the candidate. The correlation of these descriptors is plausible given that the S2 subsite, responsible for the specificity of the enzyme, is lipophilic.^7^

SMR_VSA7 is a descriptor that relates refractive properties (related to polarizability) to VSA in a given range. It may reflect aspects of the electron distribution and shape of the molecule that influence the interaction with the protein. PEOE_VSA8 is derived from the PEOE (Partial Equalization of Orbital Electronegativities) method. It combines information about the distribution of partial charges with the molecular surface. It may be useful to capture electrostatic interactions important in binding with the target.^19^

fr_bicyclic indicates the fraction of bicyclic groups present in the structure. The negative correlation may suggest that the excessive presence of bicyclic groups may decrease the activity. Additionally, MinStateIndex is one of the electrostatic indices derived from the EState (Electronic State) concept. It captures the lowest value of an index that integrates information about the electronegativity and connectivity of the atoms within the molecule, reflecting aspects of the electronic distribution. fr_ketone_Topliss counts ketone groups in the structure.^19^ The presence of ketone groups and bicyclic systems are moderately correlated with lower activity. Moreover, the descriptors most strongly correlated with pIC_50_ can be rationalized in light of cruzain’s active-site subsites. S2 subsite is lipophilic and is known to tolerate aromatic and halogenated groups. The descriptors fr_pyridine, NumAromaticHeterocycles, and fr_halogen reflect this preference and align with experimental evidence that aromatic heterocycles and halogen substituents enhance binding affinity. On the other hand, S1 and S3 subsites contribute to polar and hydrogen-bonding interactions. Descriptors such as SMR_VSA7 (related to polarizability and shape) and PEOE_VSA8 (electrostatics + surface area) are consistent with recognition in these subsites. Meanwhile, Rigid or bicyclic structures (fr_bicyclic) were negatively correlated with activity, which may reflect steric clashes and reduced adaptability within the relatively narrow S1 and S3 subsites.^9,19^

### Importance

Importance is a measure that helps assess how much each descriptor contributes to decreasing the uncertainty of a model.^30^ When a descriptor is utilized to make a split at a node, the model calculates the reduction in impurity at that node. The total importance of all descriptors is normalized to 1. Descriptors with higher importance values have a greater influence on the model’s decisions. Even if a descriptor does not show a linear correlation with pIC_50_, it can still be significant as it helps the model capture complex, nonlinear relationships within the data set.

Figure 7 shows which descriptors are most important in the RF model for predicting cruzain inhibitors. Descriptors such as fr_Ar_N, SMR_VSA7, BCUT2D_CHGLO, NumAromaticHeterocycles, and BCUT2D_MWLOW show the highest importance for the cruzain inhibitor prediction model. The descriptor fr_Ar_N counts the number of nitrogen-containing aromatic groups in the structure. It is useful for identifying the presence of pyridines, pyrazines, pyrimidines, and other nitrogen-containing aromatic heterocycles. SMR_VSA7 is a VSA-based descriptor weighted by the specific molarity refractive index (SMR). It can be useful in identifying the accessibility of different regions of the structure.^19^

**Figure 7:**
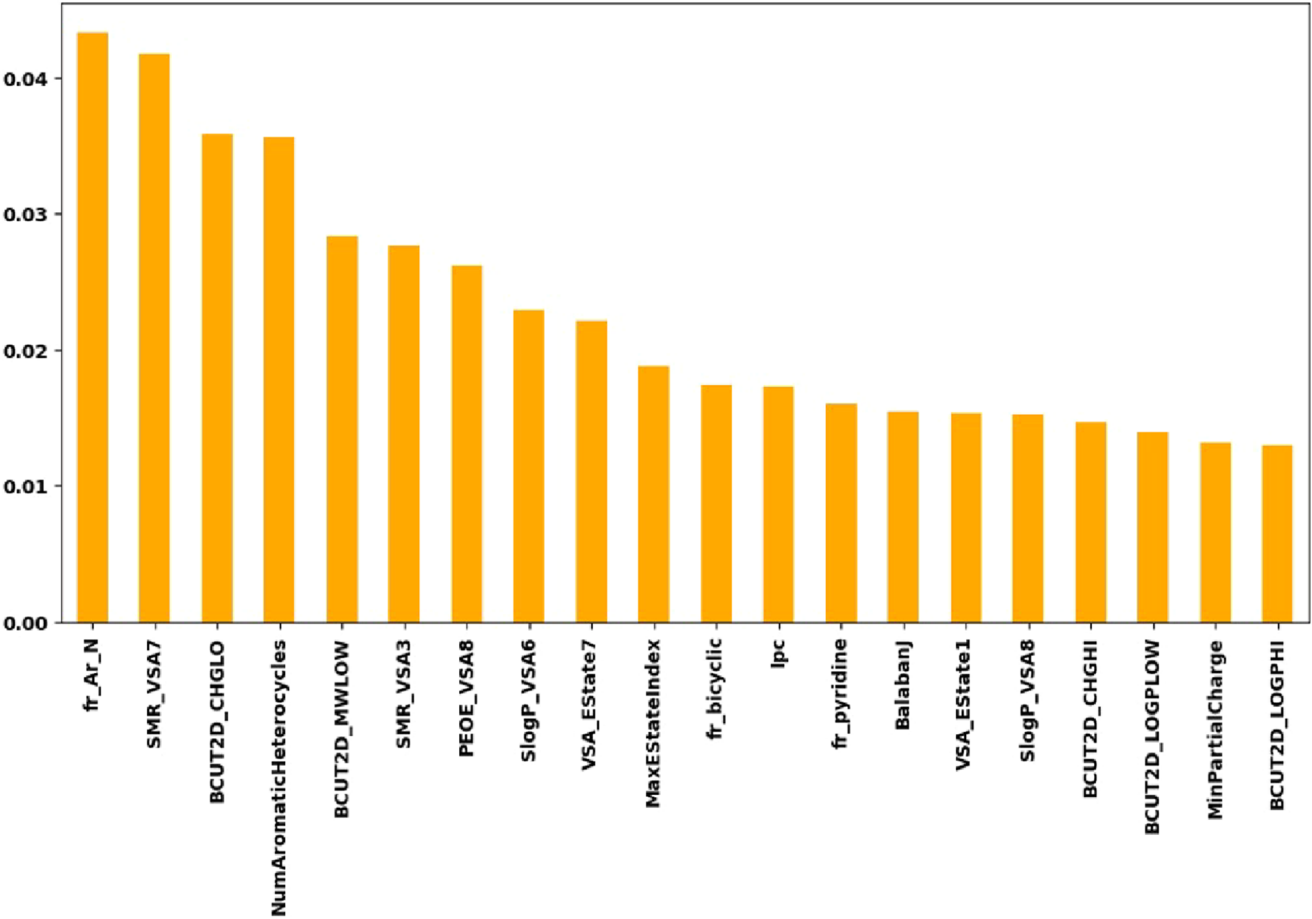
Importance of descriptors for model construction.

BCUT2D_CHGLO is a BCUT (Burden-CAS University of Texas) descriptor representing properties of the Burden matrix, in this case related to the lowest electrostatic charge (CHGLO). It indicates the presence of negatively charged regions in the molecule. BCUT2D_MWLOW is another BCUT descriptor, related to the low molecular weight within the Burden matrix. It measures the distribution of molecular weight within the molecule. The lower the value, the higher the concentration of light atoms (such as hydrogen, oxygen, nitrogen), which can influence the solubility and diffusion of the molecule.^19^ It is important to distinguish between linear correlation and feature importance in RF. Pearson’s r measures the direct linear relationship between a descriptor and activity, whereas RF importance captures the contribution of descriptors to prediction error reduction across trees, accounting for nonlinear effects and interactions. Thus, descriptors with low linear correlation may still be highly relevant in RF if they interact synergistically with others.^31^

## Conclusions

In this work, a user-friendly approach was presented for conducting in silico virtual screening of potential cruzain inhibitors by predicting their pIC_50_ values. This strategy includes customizable, ready-to-use, and shareable Jupyter notebook that assists users in performing calculations via Google Colab. This study aims to enable synthetic chemistry and bioassay researchers to predict and screen their compounds using the developed RF model.

Some relevant insights were also observed during the construction of the model. 1 - Aromaticity was shown to be a key factor in inhibitory activity. Compounds with nitrogenous aromatic rings are more likely to be more effective inhibitors. Aromatics in general also present correlation and structural relevance for an effective inhibitor. 2 - Halogenation may favor activity. The positive correlation may suggest that the introduction of halogen atoms may improve the activity of the compounds. 3 - Bicyclic or very rigid structures may decrease the inhibition efficiency of the tested candidates. 4 - Molecular accessibility and charge influence activity. The descriptors SMR_VSA7 and BCUT2D_CHGLO appear both in the importance of the RF model and in the correlation with pIC_50_. Factors such as accessible surface and partial charge play relevant roles in the interaction of the compound with the target.

## Supplementary Material



## Data Availability

The data set is freely available at GitHub: https://github.com/TropMol/TropMol-Caipora. The Colab notebook shown here is freely and publicly available at: https://colab.research.google.com/drive/1hotsXPddbJ6E0_hysLT9AqsXL-74Na-z?usp=sharing.

## Notes

Activity cliffs test, examples of decision trees used according to the selected RF parameters, general evaluation of Random Forest, Gradient Boosting, XGBoost, LightGBM, and respective tuning parameters (PDF)
