# Supplementary material for: TropMol-Caipora: A Cloud-Based Web Tool to Predict Cruzain Inhibitors by Machine Learning
Source: ACS Omega. 2026 Jan 8;11(3):4167–74. doi: 10.1021/acsomega.5c08795 (PMC12854596; doi:10.1021/acsomega.5c08795)

TropMol-Caipora: A Cloud-based web tool to predict cruzain inhibitors by machine learning

Thiago H. Doring [a]*

a) Department of Exact Sciences and Education (CEE), School of Technology, Exact Sciences and Education (CTE), Federal University of Santa Catarina (UFSC), Blumenau 89036-256, SC, Brazil.

* Corresponding author email address: thiago.doring@ufsc.br

Table of Contents

1 Tuning Parameters 3

1.1 Random Forest 3

1.2 Gradient Boosting 15

1.3 XGBoost 21

1.4 LightGBM 26

2 Activity Cliffs - Test 32

3 Examples of decision trees used according to the selected RF parameters* 34

1 Tuning Parameters

Here are shown the tuning values used in the Random Forest, Gradient Boosting, XGBoost, and LightGBM models.

1.1 Random Forest

Testing different parameter combinations for RANDOM FOREST on the normalized dataset.

Test different combinations of parameters for Random Forest on the normalized dataset (test_size=0.1)

```
param _grid_normalized = {
'n_estimators': [10, 50, 100, 110, 150],
   'max_depth': [10, 15, 20],
'min_samples_leaf': [1, 2, 4],
   'max_features': ['sqrt', 'log2']
}
```

Testing different combinations of parameters on the normalized dataset:

R^2

R^2 with n_estimators=10, max_depth=10, min_samples_leaf=1, max_features=sqrt: 0.6212

R^2 with n_estimators=10, max_depth=10, min_samples_leaf=1, max_features=log2: 0.5925

R^2 with n_estimators=10, max_depth=10, min_samples_leaf=2, max_features=sqrt: 0.6181

R^2 with n_estimators=10, max_depth=10, min_samples_leaf=2, max_features=log2: 0.6000

R^2 with n_estimators=10, max_depth=10, min_samples_leaf=4, max_features=sqrt: 0.6172

R^2 with n_estimators=10, max_depth=10, min_samples_leaf=4, max_features=log2: 0.6014

R^2 with n_estimators=10, max_depth=15, min_samples_leaf=1, max_features=sqrt: 0.6831

R^2 with n_estimators=10, max_depth=15, min_samples_leaf=1, max_features=log2: 0.6869

R^2 with n_estimators=10, max_depth=15, min_samples_leaf=2, max_features=sqrt: 0.6846

R^2 with n_estimators=10, max_depth=15, min_samples_leaf=2, max_features=log2: 0.6630

R^2 with n_estimators=10, max_depth=15, min_samples_leaf=4, max_features=sqrt: 0.6668

R^2 with n_estimators=10, max_depth=15, min_samples_leaf=4, max_features=log2: 0.6576

R^2 with n_estimators=10, max_depth=20, min_samples_leaf=1, max_features=sqrt: 0.6852

R^2 with n_estimators=10, max_depth=20, min_samples_leaf=1, max_features=log2: 0.6840

R^2 with n_estimators=10, max_depth=20, min_samples_leaf=2, max_features=sqrt: 0.6936

R^2 with n_estimators=10, max_depth=20, min_samples_leaf=2, max_features=log2: 0.6899

R^2 with n_estimators=10, max_depth=20, min_samples_leaf=4, max_features=sqrt: 0.6770

R^2 with n_estimators=10, max_depth=20, min_samples_leaf=4, max_features=log2: 0.6756

R^2 with n_estimators=50, max_depth=10, min_samples_leaf=1, max_features=sqrt: 0.6390

R^2 with n_estimators=50, max_depth=10, min_samples_leaf=1, max_features=log2: 0.6211

R^2 with n_estimators=50, max_depth=10, min_samples_leaf=2, max_features=sqrt: 0.6449

R^2 with n_estimators=50, max_depth=10, min_samples_leaf=2, max_features=log2: 0.6248

R^2 with n_estimators=50, max_depth=10, min_samples_leaf=4, max_features=sqrt: 0.6297

R^2 with n_estimators=50, max_depth=10, min_samples_leaf=4, max_features=log2: 0.6193

R^2 with n_estimators=50, max_depth=15, min_samples_leaf=1, max_features=sqrt: 0.7073

R^2 with n_estimators=50, max_depth=15, min_samples_leaf=1, max_features=log2: 0.7061

R^2 with n_estimators=50, max_depth=15, min_samples_leaf=2, max_features=sqrt: 0.7058

R^2 with n_estimators=50, max_depth=15, min_samples_leaf=2, max_features=log2: 0.6941

R^2 with n_estimators=50, max_depth=15, min_samples_leaf=4, max_features=sqrt: 0.6844

R^2 with n_estimators=50, max_depth=15, min_samples_leaf=4, max_features=log2: 0.6746

R^2 with n_estimators=50, max_depth=20, min_samples_leaf=1, max_features=sqrt: 0.7182

R^2 with n_estimators=50, max_depth=20, min_samples_leaf=1, max_features=log2: 0.7132 <-

R^2 with n_estimators=50, max_depth=20, min_samples_leaf=2, max_features=sqrt: 0.7164 <-

R^2 with n_estimators=50, max_depth=20, min_samples_leaf=2, max_features=log2: 0.7038

R^2 with n_estimators=50, max_depth=20, min_samples_leaf=4, max_features=sqrt: 0.7015

R^2 with n_estimators=50, max_depth=20, min_samples_leaf=4, max_features=log2: 0.6956

R^2 with n_estimators=100, max_depth=10, min_samples_leaf=1, max_features=sqrt: 0.6451

R^2 with n_estimators=100, max_depth=10, min_samples_leaf=1, max_features=log2: 0.6265

R^2 with n_estimators=100, max_depth=10, min_samples_leaf=2, max_features=sqrt: 0.6490

R^2 with n_estimators=100, max_depth=10, min_samples_leaf=2, max_features=log2: 0.6252

R^2 with n_estimators=100, max_depth=10, min_samples_leaf=4, max_features=sqrt: 0.6345

R^2 with n_estimators=100, max_depth=10, min_samples_leaf=4, max_features=log2: 0.6203

R^2 with n_estimators=100, max_depth=15, min_samples_leaf=1, max_features=sqrt: 0.7115

R^2 with n_estimators=100, max_depth=15, min_samples_leaf=1, max_features=log2: 0.7067

R^2 with n_estimators=100, max_depth=15, min_samples_leaf=2, max_features=sqrt: 0.7117

R^2 with n_estimators=100, max_depth=15, min_samples_leaf=2, max_features=log2: 0.6994

R^2 with n_estimators=100, max_depth=15, min_samples_leaf=4, max_features=sqrt: 0.6953

R^2 with n_estimators=100, max_depth=15, min_samples_leaf=4, max_features=log2: 0.6818

R^2 with n_estimators=100, max_depth=20, min_samples_leaf=1, max_features=sqrt: 0.7231 <-

R^2 with n_estimators=100, max_depth=20, min_samples_leaf=1, max_features=log2: 0.7194

R^2 with n_estimators=100, max_depth=20, min_samples_leaf=2, max_features=sqrt: 0.7183

R^2 with n_estimators=100, max_depth=20, min_samples_leaf=2, max_features=log2: 0.7095

R^2 with n_estimators=100, max_depth=20, min_samples_leaf=4, max_features=sqrt: 0.7038

R^2 with n_estimators=100, max_depth=20, min_samples_leaf=4, max_features=log2: 0.6961

R^2 with n_estimators=110, max_depth=10, min_samples_leaf=1, max_features=sqrt: 0.6471

R^2 with n_estimators=110, max_depth=10, min_samples_leaf=1, max_features=log2: 0.6255

R^2 with n_estimators=110, max_depth=10, min_samples_leaf=2, max_features=sqrt: 0.6486

R^2 with n_estimators=110, max_depth=10, min_samples_leaf=2, max_features=log2: 0.6237

R^2 with n_estimators=110, max_depth=10, min_samples_leaf=4, max_features=sqrt: 0.6356

R^2 with n_estimators=110, max_depth=10, min_samples_leaf=4, max_features=log2: 0.6194

R^2 with n_estimators=110, max_depth=15, min_samples_leaf=1, max_features=sqrt: 0.7114

R^2 with n_estimators=110, max_depth=15, min_samples_leaf=1, max_features=log2: 0.7059

R^2 with n_estimators=110, max_depth=15, min_samples_leaf=2, max_features=sqrt: 0.7118

R^2 with n_estimators=110, max_depth=15, min_samples_leaf=2, max_features=log2: 0.6996

R^2 with n_estimators=110, max_depth=15, min_samples_leaf=4, max_features=sqrt: 0.6950

R^2 with n_estimators=110, max_depth=15, min_samples_leaf=4, max_features=log2: 0.6806

R^2 with n_estimators=110, max_depth=20, min_samples_leaf=1, max_features=sqrt: 0.7217 <-

R^2 with n_estimators=110, max_depth=20, min_samples_leaf=1, max_features=log2: 0.7200

R^2 with n_estimators=110, max_depth=20, min_samples_leaf=2, max_features=sqrt: 0.7180

R^2 with n_estimators=110, max_depth=20, min_samples_leaf=2, max_features=log2: 0.7100

R^2 with n_estimators=110, max_depth=20, min_samples_leaf=4, max_features=sqrt: 0.7033

R^2 with n_estimators=110, max_depth=20, min_samples_leaf=4, max_features=log2: 0.6949

R^2 with n_estimators=150, max_depth=10, min_samples_leaf=1, max_features=sqrt: 0.6482

R^2 with n_estimators=150, max_depth=10, min_samples_leaf=1, max_features=log2: 0.6277

R^2 with n_estimators=150, max_depth=10, min_samples_leaf=2, max_features=sqrt: 0.6484

R^2 with n_estimators=150, max_depth=10, min_samples_leaf=2, max_features=log2: 0.6261

R^2 with n_estimators=150, max_depth=10, min_samples_leaf=4, max_features=sqrt: 0.6343

R^2 with n_estimators=150, max_depth=10, min_samples_leaf=4, max_features=log2: 0.6205

R^2 with n_estimators=150, max_depth=15, min_samples_leaf=1, max_features=sqrt: 0.7127

R^2 with n_estimators=150, max_depth=15, min_samples_leaf=1, max_features=log2: 0.7063

R^2 with n_estimators=150, max_depth=15, min_samples_leaf=2, max_features=sqrt: 0.7110

R^2 with n_estimators=150, max_depth=15, min_samples_leaf=2, max_features=log2: 0.7008

R^2 with n_estimators=150, max_depth=15, min_samples_leaf=4, max_features=sqrt: 0.6955

R^2 with n_estimators=150, max_depth=15, min_samples_leaf=4, max_features=log2: 0.6817

R^2 with n_estimators=150, max_depth=20, min_samples_leaf=1, max_features=sqrt: 0.7226

R^2 with n_estimators=150, max_depth=20, min_samples_leaf=1, max_features=log2: 0.7209

R^2 with n_estimators=150, max_depth=20, min_samples_leaf=2, max_features=sqrt: 0.7186

R^2 with n_estimators=150, max_depth=20, min_samples_leaf=2, max_features=log2: 0.7107

R^2 with n_estimators=150, max_depth=20, min_samples_leaf=4, max_features=sqrt: 0.7055

R^2 with n_estimators=150, max_depth=20, min_samples_leaf=4, max_features=log2: 0.6943

Best parameters found in the normalized dataset:

{'n_estimators': 100, 'max_depth': 20, 'min_samples_leaf': 1, 'max_features': 'sqrt'} 0.7231

```
# Test different combinations of parameters for Random Forest on the normalized dataset (test_size=0.1)

param _grid_normalized = {
'n_estimators': [50, 100, 110],
   'max_depth': [15, 20, 25, 30, 35],
'min_samples_leaf': [1],
   'max_features': ['sqrt', 'log2']
}
```

Testing different combinations of parameters on the normalized dataset:

R^2

R^2 with n_estimators=50, max_depth=15, min_samples_leaf=1, max_features=sqrt: 0.7060
R^2 with n_estimators=50, max_depth=15, min_samples_leaf=1, max_features=log2: 0.6975
R^2 with n_estimators=50, max_depth=20, min_samples_leaf=1, max_features=sqrt: 0.7172
R^2 with n_estimators=50, max_depth=20, min_samples_leaf=1, max_features=log2: 0.7112
R^2 with n_estimators=50, max_depth=25, min_samples_leaf=1, max_features=sqrt: 0.7144
R^2 with n_estimators=50, max_depth=25, min_samples_leaf=1, max_features=log2: 0.7183
R^2 with n_estimators=50, max_depth=30, min_samples_leaf=1, max_features=sqrt: 0.7237
R^2 with n_estimators=50, max_depth=30, min_samples_leaf=1, max_features=log2: 0.7105
R^2 with n_estimators=50, max_depth=35, min_samples_leaf=1, max_features=sqrt: 0.7179
R^2 with n_estimators=50, max_depth=35, min_samples_leaf=1, max_features=log2: 0.7128
R^2 with n_estimators=100, max_depth=15, min_samples_leaf=1, max_features=sqrt: 0.7115
R^2 with n_estimators=100, max_depth=15, min_samples_leaf=1, max_features=log2: 0.7068
R^2 with n_estimators=100, max_depth=20, min_samples_leaf=1, max_features=sqrt: 0.7224
R^2 with n_estimators=100, max_depth=20, min_samples_leaf=1, max_features=log2: 0.7138
R^2 with n_estimators=100, max_depth=25, min_samples_leaf=1, max_features=sqrt: 0.7178
R^2 with n_estimators=100, max_depth=25, min_samples_leaf=1, max_features=log2: 0.7189
R^2 with n_estimators=100, max_depth=30, min_samples_leaf=1, max_features=sqrt: 0.7247 <--
R^2 with n_estimators=100, max_depth=30, min_samples_leaf=1, max_features=log2: 0.7130
R^2 with n_estimators=100, max_depth=35, min_samples_leaf=1, max_features=sqrt: 0.7203
R^2 with n_estimators=100, max_depth=35, min_samples_leaf=1, max_features=log2: 0.7152
R^2 with n_estimators=110, max_depth=15, min_samples_leaf=1, max_features=sqrt: 0.7119
R^2 with n_estimators=110, max_depth=15, min_samples_leaf=1, max_features=log2: 0.7062
R^2 with n_estimators=110, max_depth=20, min_samples_leaf=1, max_features=sqrt: 0.7231
R^2 with n_estimators=110, max_depth=20, min_samples_leaf=1, max_features=log2: 0.7148

R^2 with n_estimators=110, max_depth=25, min_samples_leaf=1, max_features=sqrt: 0.7175

R^2 with n_estimators=110, max_depth=25, min_samples_leaf=1, max_features=log2: 0.7194

R^2 with n_estimators=110, max_depth=30, min_samples_leaf=1, max_features=sqrt: 0.7226

R^2 with n_estimators=110, max_depth=30, min_samples_leaf=1, max_features=log2: 0.7134

R^2 with n_estimators=110, max_depth=35, min_samples_leaf=1, max_features=sqrt: 0.7204

R^2 with n_estimators=110, max_depth=35, min_samples_leaf=1, max_features=log2: 0.7150

Best parameters found in the normalized dataset:

{'n_estimators': 100, 'max_depth': 30, 'min_samples_leaf': 1, 'max_features': 'sqrt'} 0.7247

Test different combinations of parameters for Random Forest on the normalized dataset (test_size=0.05)

```
param _grid_normalized = {
'n_estimators': [10, 50, 100, 110, 150],
   'max_depth': [10, 15, 20],
'min_samples_leaf': [1, 2, 4],
   'max_features': ['sqrt', 'log2']
}
```

Testing different combinations of parameters on the normalized dataset:

R^2

R^2 with n_estimators=10, max_depth=10, min_samples_leaf=1, max_features=sqrt: 0.6353
R^2 with n_estimators=10, max_depth=10, min_samples_leaf=1, max_features=log2: 0.6244
R^2 with n_estimators=10, max_depth=10, min_samples_leaf=2, max_features=sqrt: 0.6253
R^2 with n_estimators=10, max_depth=10, min_samples_leaf=2, max_features=log2: 0.6136
R^2 with n_estimators=10, max_depth=10, min_samples_leaf=4, max_features=sqrt: 0.6159
R^2 with n_estimators=10, max_depth=10, min_samples_leaf=4, max_features=log2: 0.5940
R^2 with n_estimators=10, max_depth=15, min_samples_leaf=1, max_features=sqrt: 0.6900
R^2 with n_estimators=10, max_depth=15, min_samples_leaf=1, max_features=log2: 0.6680
R^2 with n_estimators=10, max_depth=15, min_samples_leaf=2, max_features=sqrt: 0.7077
R^2 with n_estimators=10, max_depth=15, min_samples_leaf=2, max_features=log2: 0.6873
R^2 with n_estimators=10, max_depth=15, min_samples_leaf=4, max_features=sqrt: 0.6800
R^2 with n_estimators=10, max_depth=15, min_samples_leaf=4, max_features=log2: 0.6541
R^2 with n_estimators=10, max_depth=20, min_samples_leaf=1, max_features=sqrt: 0.6952
R^2 with n_estimators=10, max_depth=20, min_samples_leaf=1, max_features=log2: 0.6855
R^2 with n_estimators=10, max_depth=20, min_samples_leaf=2, max_features=sqrt: 0.6968
R^2 with n_estimators=10, max_depth=20, min_samples_leaf=2, max_features=log2: 0.6968
R^2 with n_estimators=10, max_depth=20, min_samples_leaf=4, max_features=sqrt: 0.6874
R^2 with n_estimators=10, max_depth=20, min_samples_leaf=4, max_features=log2: 0.6800
R^2 with n_estimators=50, max_depth=10, min_samples_leaf=1, max_features=sqrt: 0.6438
R^2 with n_estimators=50, max_depth=10, min_samples_leaf=1, max_features=log2: 0.6355
R^2 with n_estimators=50, max_depth=10, min_samples_leaf=2, max_features=sqrt: 0.6366
R^2 with n_estimators=50, max_depth=10, min_samples_leaf=2, max_features=log2: 0.6357
R^2 with n_estimators=50, max_depth=10, min_samples_leaf=4, max_features=sqrt: 0.6370
R^2 with n_estimators=50, max_depth=10, min_samples_leaf=4, max_features=log2: 0.6114
R^2 with n_estimators=50, max_depth=15, min_samples_leaf=1, max_features=sqrt: 0.7177

R^2 with n_estimators=50, max_depth=15, min_samples_leaf=1, max_features=log2: 0.6998

R^2 with n_estimators=50, max_depth=15, min_samples_leaf=2, max_features=sqrt: 0.7108

R^2 with n_estimators=50, max_depth=15, min_samples_leaf=2, max_features=log2: 0.7115

R^2 with n_estimators=50, max_depth=15, min_samples_leaf=4, max_features=sqrt: 0.6930

R^2 with n_estimators=50, max_depth=15, min_samples_leaf=4, max_features=log2: 0.6866

R^2 with n_estimators=50, max_depth=20, min_samples_leaf=1, max_features=sqrt: 0.7246

R^2 with n_estimators=50, max_depth=20, min_samples_leaf=1, max_features=log2: 0.7170

R^2 with n_estimators=50, max_depth=20, min_samples_leaf=2, max_features=sqrt: 0.7113

R^2 with n_estimators=50, max_depth=20, min_samples_leaf=2, max_features=log2: 0.7234 <-

R^2 with n_estimators=50, max_depth=20, min_samples_leaf=4, max_features=sqrt: 0.7077

R^2 with n_estimators=50, max_depth=20, min_samples_leaf=4, max_features=log2: 0.7030

R^2 with n_estimators=100, max_depth=10, min_samples_leaf=1, max_features=sqrt: 0.6519

R^2 with n_estimators=100, max_depth=10, min_samples_leaf=1, max_features=log2: 0.6391

R^2 with n_estimators=100, max_depth=10, min_samples_leaf=2, max_features=sqrt: 0.6495

R^2 with n_estimators=100, max_depth=10, min_samples_leaf=2, max_features=log2: 0.6370

R^2 with n_estimators=100, max_depth=10, min_samples_leaf=4, max_features=sqrt: 0.6427

R^2 with n_estimators=100, max_depth=10, min_samples_leaf=4, max_features=log2: 0.6216

R^2 with n_estimators=100, max_depth=15, min_samples_leaf=1, max_features=sqrt: 0.7190

R^2 with n_estimators=100, max_depth=15, min_samples_leaf=1, max_features=log2: 0.7081

R^2 with n_estimators=100, max_depth=15, min_samples_leaf=2, max_features=sqrt: 0.7137

R^2 with n_estimators=100, max_depth=15, min_samples_leaf=2, max_features=log2: 0.7110

R^2 with n_estimators=100, max_depth=15, min_samples_leaf=4, max_features=sqrt: 0.7017

R^2 with n_estimators=100, max_depth=15, min_samples_leaf=4, max_features=log2: 0.6868

R^2 with n_estimators=100, max_depth=20, min_samples_leaf=1, max_features=sqrt: 0.7263 <-

R^2 with n_estimators=100, max_depth=20, min_samples_leaf=1, max_features=log2: 0.7228

R^2 with n_estimators=100, max_depth=20, min_samples_leaf=2, max_features=sqrt: 0.7211

R^2 with n_estimators=100, max_depth=20, min_samples_leaf=2, max_features=log2: 0.7214

R^2 with n_estimators=100, max_depth=20, min_samples_leaf=4, max_features=sqrt: 0.7114

R^2 with n_estimators=100, max_depth=20, min_samples_leaf=4, max_features=log2: 0.7029

R^2 with n_estimators=110, max_depth=10, min_samples_leaf=1, max_features=sqrt: 0.6522

R^2 with n_estimators=110, max_depth=10, min_samples_leaf=1, max_features=log2: 0.6390

R^2 with n_estimators=110, max_depth=10, min_samples_leaf=2, max_features=sqrt: 0.6497

R^2 with n_estimators=110, max_depth=10, min_samples_leaf=2, max_features=log2: 0.6377

R^2 with n_estimators=110, max_depth=10, min_samples_leaf=4, max_features=sqrt: 0.6436

R^2 with n_estimators=110, max_depth=10, min_samples_leaf=4, max_features=log2: 0.6206

R^2 with n_estimators=110, max_depth=15, min_samples_leaf=1, max_features=sqrt: 0.7196

R^2 with n_estimators=110, max_depth=15, min_samples_leaf=1, max_features=log2: 0.7099

R^2 with n_estimators=110, max_depth=15, min_samples_leaf=2, max_features=sqrt: 0.7145

R^2 with n_estimators=110, max_depth=15, min_samples_leaf=2, max_features=log2: 0.7121

R^2 with n_estimators=110, max_depth=15, min_samples_leaf=4, max_features=sqrt: 0.7021

R^2 with n_estimators=110, max_depth=15, min_samples_leaf=4, max_features=log2: 0.6876

R^2 with n_estimators=110, max_depth=20, min_samples_leaf=1, max_features=sqrt: 0.7279 <-

R^2 with n_estimators=110, max_depth=20, min_samples_leaf=1, max_features=log2: 0.7249 <-

R^2 with n_estimators=110, max_depth=20, min_samples_leaf=2, max_features=sqrt: 0.7221

R^2 with n_estimators=110, max_depth=20, min_samples_leaf=2, max_features=log2: 0.7213

R^2 with n_estimators=110, max_depth=20, min_samples_leaf=4, max_features=sqrt: 0.7132

R^2 with n_estimators=110, max_depth=20, min_samples_leaf=4, max_features=log2: 0.7043

R^2 with n_estimators=150, max_depth=10, min_samples_leaf=1, max_features=sqrt: 0.6519

R^2 with n_estimators=150, max_depth=10, min_samples_leaf=1, max_features=log2: 0.6346

R^2 with n_estimators=150, max_depth=10, min_samples_leaf=2, max_features=sqrt: 0.6488

R^2 with n_estimators=150, max_depth=10, min_samples_leaf=2, max_features=log2: 0.6348

R^2 with n_estimators=150, max_depth=10, min_samples_leaf=4, max_features=sqrt: 0.6427

R^2 with n_estimators=150, max_depth=10, min_samples_leaf=4, max_features=log2: 0.6235

R^2 with n_estimators=150, max_depth=15, min_samples_leaf=1, max_features=sqrt: 0.7200

R^2 with n_estimators=150, max_depth=15, min_samples_leaf=1, max_features=log2: 0.7110

R^2 with n_estimators=150, max_depth=15, min_samples_leaf=2, max_features=sqrt: 0.7154

R^2 with n_estimators=150, max_depth=15, min_samples_leaf=2, max_features=log2: 0.7116

R^2 with n_estimators=150, max_depth=15, min_samples_leaf=4, max_features=sqrt: 0.7014

R^2 with n_estimators=150, max_depth=15, min_samples_leaf=4, max_features=log2: 0.6855

R^2 with n_estimators=150, max_depth=20, min_samples_leaf=1, max_features=sqrt: 0.7274

R^2 with n_estimators=150, max_depth=20, min_samples_leaf=1, max_features=log2: 0.7266

R^2 with n_estimators=150, max_depth=20, min_samples_leaf=2, max_features=sqrt: 0.7237

R^2 with n_estimators=150, max_depth=20, min_samples_leaf=2, max_features=log2: 0.7221

R^2 with n_estimators=150, max_depth=20, min_samples_leaf=4, max_features=sqrt: 0.7109

R^2 with n_estimators=150, max_depth=20, min_samples_leaf=4, max_features=log2: 0.7047

Best parameters found in the normalized dataset:

{'n_estimators': 110, 'max_depth': 20, 'min_samples_leaf': 1, 'max_features': 'sqrt'} 0.7279

Test different combinations of parameters for Random Forest on the normalized dataset (test_size=0.05)

param _grid_normalized = {
'n_estimators': [50, 100, 110],
'max_depth': [15, 20, 25, 30, 35],
'min_samples_leaf': [1],
'max_features': ['sqrt', 'log2']
}

Testing different combinations of parameters on the normalized dataset:

R^2

R^2 with n_estimators=50, max_depth=15, min_samples_leaf=1, max_features=sqrt: 0.7177
R^2 with n_estimators=50, max_depth=15, min_samples_leaf=1, max_features=log2: 0.6998
R^2 with n_estimators=50, max_depth=20, min_samples_leaf=1, max_features=sqrt: 0.7246
R^2 with n_estimators=50, max_depth=20, min_samples_leaf=1, max_features=log2: 0.7170
R^2 with n_estimators=50, max_depth=25, min_samples_leaf=1, max_features=sqrt: 0.7278
R^2 with n_estimators=50, max_depth=25, min_samples_leaf=1, max_features=log2: 0.7169
R^2 with n_estimators=50, max_depth=30, min_samples_leaf=1, max_features=sqrt: 0.7209
R^2 with n_estimators=50, max_depth=30, min_samples_leaf=1, max_features=log2: 0.7138
R^2 with n_estimators=50, max_depth=35, min_samples_leaf=1, max_features=sqrt: 0.7217
R^2 with n_estimators=50, max_depth=35, min_samples_leaf=1, max_features=log2: 0.7128
R^2 with n_estimators=100, max_depth=15, min_samples_leaf=1, max_features=sqrt: 0.7190
R^2 with n_estimators=100, max_depth=15, min_samples_leaf=1, max_features=log2: 0.7081
R^2 with n_estimators=100, max_depth=20, min_samples_leaf=1, max_features=sqrt: 0.7263
R^2 with n_estimators=100, max_depth=20, min_samples_leaf=1, max_features=log2: 0.7228
R^2 with n_estimators=100, max_depth=25, min_samples_leaf=1, max_features=sqrt: 0.7322 <-
R^2 with n_estimators=100, max_depth=25, min_samples_leaf=1, max_features=log2: 0.7217
R^2 with n_estimators=100, max_depth=30, min_samples_leaf=1, max_features=sqrt: 0.7296 <--
R^2 with n_estimators=100, max_depth=30, min_samples_leaf=1, max_features=log2: 0.7187
R^2 with n_estimators=100, max_depth=35, min_samples_leaf=1, max_features=sqrt: 0.7269
R^2 with n_estimators=100, max_depth=35, min_samples_leaf=1, max_features=log2: 0.7207
R^2 with n_estimators=110, max_depth=15, min_samples_leaf=1, max_features=sqrt: 0.7196
R^2 with n_estimators=110, max_depth=15, min_samples_leaf=1, max_features=log2: 0.7099
R^2 with n_estimators=110, max_depth=20, min_samples_leaf=1, max_features=sqrt: 0.7279

R^2 with n_estimators=110, max_depth=20, min_samples_leaf=1, max_features=log2: 0.7249

R^2 with n_estimators=110, max_depth=25, min_samples_leaf=1, max_features=sqrt: 0.7329 <-

R^2 with n_estimators=110, max_depth=25, min_samples_leaf=1, max_features=log2: 0.7224

R^2 with n_estimators=110, max_depth=30, min_samples_leaf=1, max_features=sqrt: 0.7294

R^2 with n_estimators=110, max_depth=30, min_samples_leaf=1, max_features=log2: 0.7205

R^2 with n_estimators=110, max_depth=35, min_samples_leaf=1, max_features=sqrt: 0.7269

R^2 with n_estimators=110, max_depth=35, min_samples_leaf=1, max_features=log2: 0.7217

Best parameters found in the normalized dataset:

{'n_estimators': 110, 'max_depth': 25, 'min_samples_leaf': 1, 'max_features': 'sqrt'} 0.7329

```
# Testing different parameter combinations for Random Forest with PCA
param_grid_pca = {
    'n_estimators': [200, 300, 500, 1000, 2000],
    'max_depth': [25, 30, 35, 40],
    'min_samples_leaf': [1, 2],
    'max_features': ['sqrt']
}

pca = PCA(n_components=0.95)
X_train_pca = pca.fit_transform(X_train_scaled)
X_test_pca = pca.transform(X_test_scaled)
```

```
Testing different parameter combinations with PCA:
R² with n_estimators=200, max_depth=25, min_samples_leaf=1, max_features=sqrt: 0.5717
R² with n_estimators=200, max_depth=25, min_samples_leaf=2, max_features=sqrt: 0.5691
R² with n_estimators=200, max_depth=30, min_samples_leaf=1, max_features=sqrt: 0.5702
R² with n_estimators=200, max_depth=30, min_samples_leaf=2, max_features=sqrt: 0.5686
R² with n_estimators=200, max_depth=35, min_samples_leaf=1, max_features=sqrt: 0.5726
R² with n_estimators=200, max_depth=35, min_samples_leaf=2, max_features=sqrt: 0.5683
R² with n_estimators=200, max_depth=40, min_samples_leaf=1, max_features=sqrt: 0.5721
R² with n_estimators=200, max_depth=40, min_samples_leaf=2, max_features=sqrt: 0.5683
R² with n_estimators=300, max_depth=25, min_samples_leaf=1, max_features=sqrt: 0.5735
R² with n_estimators=300, max_depth=25, min_samples_leaf=2, max_features=sqrt: 0.5719
R² with n_estimators=300, max_depth=30, min_samples_leaf=1, max_features=sqrt: 0.5701
R² with n_estimators=300, max_depth=30, min_samples_leaf=2, max_features=sqrt: 0.5714
R² with n_estimators=300, max_depth=35, min_samples_leaf=1, max_features=sqrt: 0.5713
R² with n_estimators=300, max_depth=35, min_samples_leaf=2, max_features=sqrt: 0.5711
R² with n_estimators=300, max_depth=40, min_samples_leaf=1, max_features=sqrt: 0.5711
R² with n_estimators=300, max_depth=40, min_samples_leaf=2, max_features=sqrt: 0.5711
R² with n_estimators=500, max_depth=25, min_samples_leaf=1, max_features=sqrt: 0.5743
R² with n_estimators=500, max_depth=25, min_samples_leaf=2, max_features=sqrt: 0.5735
R² with n_estimators=500, max_depth=30, min_samples_leaf=1, max_features=sqrt: 0.5721
R² with n_estimators=500, max_depth=30, min_samples_leaf=2, max_features=sqrt: 0.5725
R² with n_estimators=500, max_depth=35, min_samples_leaf=1, max_features=sqrt: 0.5728
R² with n_estimators=500, max_depth=35, min_samples_leaf=2, max_features=sqrt: 0.5724
R² with n_estimators=500, max_depth=40, min_samples_leaf=1, max_features=sqrt: 0.5727
R² with n_estimators=500, max_depth=40, min_samples_leaf=2, max_features=sqrt: 0.5724
```

R^2 with n_estimators=1000, max_depth=25, min_samples_leaf=1, max_features=sqrt: 0.5742

R^2 with n_estimators=1000, max_depth=25, min_samples_leaf=2, max_features=sqrt: 0.5743

R^2 with n_estimators=1000, max_depth=30, min_samples_leaf=1, max_features=sqrt: 0.5729

R^2 with n_estimators=1000, max_depth=30, min_samples_leaf=2, max_features=sqrt: 0.5749

R^2 with n_estimators=1000, max_depth=35, min_samples_leaf=1, max_features=sqrt: 0.5739

R^2 with n_estimators=1000, max_depth=35, min_samples_leaf=2, max_features=sqrt: 0.5748

R^2 with n_estimators=1000, max_depth=40, min_samples_leaf=1, max_features=sqrt: 0.5738

R^2 with n_estimators=1000, max_depth=40, min_samples_leaf=2, max_features=sqrt: 0.5748

R^2 with n_estimators=2000, max_depth=25, min_samples_leaf=1, max_features=sqrt: 0.5735

R^2 with n_estimators=2000, max_depth=25, min_samples_leaf=2, max_features=sqrt: 0.5744

R^2 with n_estimators=2000, max_depth=30, min_samples_leaf=1, max_features=sqrt: 0.5741

R^2 with n_estimators=2000, max_depth=30, min_samples_leaf=2, max_features=sqrt: 0.5750

R^2 with n_estimators=2000, max_depth=35, min_samples_leaf=1, max_features=sqrt: 0.5747

R^2 with n_estimators=2000, max_depth=35, min_samples_leaf=2, max_features=sqrt: 0.5748

R^2 with n_estimators=2000, max_depth=40, min_samples_leaf=1, max_features=sqrt: 0.5746

R^2 with n_estimators=2000, max_depth=40, min_samples_leaf=2, max_features=sqrt: 0.5748

Best parameters found with PCA:
{'n_estimators': 2000, 'max_depth': 30, 'min_samples_leaf': 2, 'max_features': 'sqrt'}

X_train, X_test, y_train, y_test = train_test_split(X, y, test_size=0.15, random_state=42)
R^2 of the final model: 0.6717
RMSE of the final model: 0.6694

cv_scores_pca = cross_val_score(rf_model_pca, X_train_pca, y_train, cv=5, scoring='r2')
Mean R^2 with PCA: 0.5767
Standard deviation of R^2 with PCA: 0.0098

1.2 Gradient Boosting

```
# Testing different parameter combinations for Gradient Boosting on the normalized dataset
param_grid_normalized = {
    'n_estimators': [10, 50, 100, 110, 150],
    'max_depth': [3, 5, 7, 10],
    'learning_rate': [0.01, 0.05, 0.1],
    'min_samples_leaf': [1, 2, 4]
}
```

Testing different parameter combinations on the normalized dataset:

R^2 with n_estimators=10, max_depth=3, learning_rate=0.01, min_samples_leaf=1: 0.0660

R^2 with n_estimators=10, max_depth=3, learning_rate=0.01, min_samples_leaf=2: 0.0660

R^2 with n_estimators=10, max_depth=3, learning_rate=0.01, min_samples_leaf=4: 0.0660

R^2 with n_estimators=10, max_depth=3, learning_rate=0.05, min_samples_leaf=1: 0.2501

R^2 with n_estimators=10, max_depth=3, learning_rate=0.05, min_samples_leaf=2: 0.2501

R^2 with n_estimators=10, max_depth=3, learning_rate=0.05, min_samples_leaf=4: 0.2501

R^2 with n_estimators=10, max_depth=3, learning_rate=0.1, min_samples_leaf=1: 0.3603

R^2 with n_estimators=10, max_depth=3, learning_rate=0.1, min_samples_leaf=2: 0.3603

R^2 with n_estimators=10, max_depth=3, learning_rate=0.1, min_samples_leaf=4: 0.3601

R^2 with n_estimators=10, max_depth=5, learning_rate=0.01, min_samples_leaf=1: 0.0838

R^2 with n_estimators=10, max_depth=5, learning_rate=0.01, min_samples_leaf=2: 0.0838

R^2 with n_estimators=10, max_depth=5, learning_rate=0.01, min_samples_leaf=4: 0.0833

R^2 with n_estimators=10, max_depth=5, learning_rate=0.05, min_samples_leaf=1: 0.3215

R^2 with n_estimators=10, max_depth=5, learning_rate=0.05, min_samples_leaf=2: 0.3215

R^2 with n_estimators=10, max_depth=5, learning_rate=0.05, min_samples_leaf=4: 0.3206

R^2 with n_estimators=10, max_depth=5, learning_rate=0.1, min_samples_leaf=1: 0.4569

R^2 with n_estimators=10, max_depth=5, learning_rate=0.1, min_samples_leaf=2: 0.4573

R^2 with n_estimators=10, max_depth=5, learning_rate=0.1, min_samples_leaf=4: 0.4559

R^2 with n_estimators=10, max_depth=7, learning_rate=0.01, min_samples_leaf=1: 0.0994

R^2 with n_estimators=10, max_depth=7, learning_rate=0.01, min_samples_leaf=2: 0.0993

R^2 with n_estimators=10, max_depth=7, learning_rate=0.01, min_samples_leaf=4: 0.0981

R^2 with n_estimators=10, max_depth=7, learning_rate=0.05, min_samples_leaf=1: 0.3729

R^2 with n_estimators=10, max_depth=7, learning_rate=0.05, min_samples_leaf=2: 0.3732

R^2 with n_estimators=10, max_depth=7, learning_rate=0.05, min_samples_leaf=4: 0.3690

R^2 with n_estimators=10, max_depth=7, learning_rate=0.1, min_samples_leaf=1: 0.5277

R^2 with n_estimators=10, max_depth=7, learning_rate=0.1, min_samples_leaf=2: 0.5311

R^2 with n_estimators=10, max_depth=7, learning_rate=0.1, min_samples_leaf=4: 0.5254

R^2 with n_estimators=10, max_depth=10, learning_rate=0.01, min_samples_leaf=1: 0.1172

R^2 with n_estimators=10, max_depth=10, learning_rate=0.01, min_samples_leaf=2: 0.1167

R^2 with n_estimators=10, max_depth=10, learning_rate=0.01, min_samples_leaf=4: 0.1170

R^2 with n_estimators=10, max_depth=10, learning_rate=0.05, min_samples_leaf=1: 0.4294

R^2 with n_estimators=10, max_depth=10, learning_rate=0.05, min_samples_leaf=2: 0.4296

R^2 with n_estimators=10, max_depth=10, learning_rate=0.05, min_samples_leaf=4: 0.4312

R^2 with n_estimators=10, max_depth=10, learning_rate=0.1, min_samples_leaf=1: 0.5911

R^2 with n_estimators=10, max_depth=10, learning_rate=0.1, min_samples_leaf=2: 0.5914

R^2 with n_estimators=10, max_depth=10, learning_rate=0.1, min_samples_leaf=4: 0.5868

R^2 with n_estimators=50, max_depth=3, learning_rate=0.01, min_samples_leaf=1: 0.2456

R^2 with n_estimators=50, max_depth=3, learning_rate=0.01, min_samples_leaf=2: 0.2456

R^2 with n_estimators=50, max_depth=3, learning_rate=0.01, min_samples_leaf=4: 0.2456

R^2 with n_estimators=50, max_depth=3, learning_rate=0.05, min_samples_leaf=1: 0.4665

R^2 with n_estimators=50, max_depth=3, learning_rate=0.05, min_samples_leaf=2: 0.4665

R^2 with n_estimators=50, max_depth=3, learning_rate=0.05, min_samples_leaf=4: 0.4677

R^2 with n_estimators=50, max_depth=3, learning_rate=0.1, min_samples_leaf=1: 0.5267

R^2 with n_estimators=50, max_depth=3, learning_rate=0.1, min_samples_leaf=2: 0.5266

R^2 with n_estimators=50, max_depth=3, learning_rate=0.1, min_samples_leaf=4: 0.5255

R^2 with n_estimators=50, max_depth=5, learning_rate=0.01, min_samples_leaf=1: 0.3174

R^2 with n_estimators=50, max_depth=5, learning_rate=0.01, min_samples_leaf=2: 0.3176

R^2 with n_estimators=50, max_depth=5, learning_rate=0.01, min_samples_leaf=4: 0.3167

R^2 with n_estimators=50, max_depth=5, learning_rate=0.05, min_samples_leaf=1: 0.5775

R^2 with n_estimators=50, max_depth=5, learning_rate=0.05, min_samples_leaf=2: 0.5803

R^2 with n_estimators=50, max_depth=5, learning_rate=0.05, min_samples_leaf=4: 0.5799

R^2 with n_estimators=50, max_depth=5, learning_rate=0.1, min_samples_leaf=1: 0.6262

R^2 with n_estimators=50, max_depth=5, learning_rate=0.1, min_samples_leaf=2: 0.6318

R^2 with n_estimators=50, max_depth=5, learning_rate=0.1, min_samples_leaf=4: 0.6276

R^2 with n_estimators=50, max_depth=7, learning_rate=0.01, min_samples_leaf=1: 0.3681

R^2 with n_estimators=50, max_depth=7, learning_rate=0.01, min_samples_leaf=2: 0.3679

R^2 with n_estimators=50, max_depth=7, learning_rate=0.01, min_samples_leaf=4: 0.3648

R^2 with n_estimators=50, max_depth=7, learning_rate=0.05, min_samples_leaf=1: 0.6516

R^2 with n_estimators=50, max_depth=7, learning_rate=0.05, min_samples_leaf=2: 0.6577

R^2 with n_estimators=50, max_depth=7, learning_rate=0.05, min_samples_leaf=4: 0.6528

R^2 with n_estimators=50, max_depth=7, learning_rate=0.1, min_samples_leaf=1: 0.6769

R^2 with n_estimators=50, max_depth=7, learning_rate=0.1, min_samples_leaf=2: 0.6831

R^2 with n_estimators=50, max_depth=7, learning_rate=0.1, min_samples_leaf=4: 0.6771

R^2 with n_estimators=50, max_depth=10, learning_rate=0.01, min_samples_leaf=1: 0.4283

R^2 with n_estimators=50, max_depth=10, learning_rate=0.01, min_samples_leaf=2: 0.4277

R^2 with n_estimators=50, max_depth=10, learning_rate=0.01, min_samples_leaf=4: 0.4292

R^2 with n_estimators=50, max_depth=10, learning_rate=0.05, min_samples_leaf=1: 0.6784

R^2 with n_estimators=50, max_depth=10, learning_rate=0.05, min_samples_leaf=2: 0.6781

R^2 with n_estimators=50, max_depth=10, learning_rate=0.05, min_samples_leaf=4: 0.6853

R^2 with n_estimators=50, max_depth=10, learning_rate=0.1, min_samples_leaf=1: 0.6740

R^2 with n_estimators=50, max_depth=10, learning_rate=0.1, min_samples_leaf=2: 0.6726

R^2 with n_estimators=50, max_depth=10, learning_rate=0.1, min_samples_leaf=4: 0.6735

R^2 with n_estimators=100, max_depth=3, learning_rate=0.01, min_samples_leaf=1: 0.3513

R^2 with n_estimators=100, max_depth=3, learning_rate=0.01, min_samples_leaf=2: 0.3513

R^2 with n_estimators=100, max_depth=3, learning_rate=0.01, min_samples_leaf=4: 0.3513

R^2 with n_estimators=100, max_depth=3, learning_rate=0.05, min_samples_leaf=1: 0.5257

R^2 with n_estimators=100, max_depth=3, learning_rate=0.05, min_samples_leaf=2: 0.5256

R^2 with n_estimators=100, max_depth=3, learning_rate=0.05, min_samples_leaf=4: 0.5238

R^2 with n_estimators=100, max_depth=3, learning_rate=0.1, min_samples_leaf=1: 0.5734

R^2 with n_estimators=100, max_depth=3, learning_rate=0.1, min_samples_leaf=2: 0.5730

R^2 with n_estimators=100, max_depth=3, learning_rate=0.1, min_samples_leaf=4: 0.5755

R^2 with n_estimators=100, max_depth=5, learning_rate=0.01, min_samples_leaf=1: 0.4514

R^2 with n_estimators=100, max_depth=5, learning_rate=0.01, min_samples_leaf=2: 0.4526

R^2 with n_estimators=100, max_depth=5, learning_rate=0.01, min_samples_leaf=4: 0.4513

R^2 with n_estimators=100, max_depth=5, learning_rate=0.05, min_samples_leaf=1: 0.6268

R^2 with n_estimators=100, max_depth=5, learning_rate=0.05, min_samples_leaf=2: 0.6259

R^2 with n_estimators=100, max_depth=5, learning_rate=0.05, min_samples_leaf=4: 0.6279

R^2 with n_estimators=100, max_depth=5, learning_rate=0.1, min_samples_leaf=1: 0.6521

R^2 with n_estimators=100, max_depth=5, learning_rate=0.1, min_samples_leaf=2: 0.6609

R^2 with n_estimators=100, max_depth=5, learning_rate=0.1, min_samples_leaf=4: 0.6586

R^2 with n_estimators=100, max_depth=7, learning_rate=0.01, min_samples_leaf=1: 0.5227

R^2 with n_estimators=100, max_depth=7, learning_rate=0.01, min_samples_leaf=2: 0.5247

R^2 with n_estimators=100, max_depth=7, learning_rate=0.01, min_samples_leaf=4: 0.5227

R^2 with n_estimators=100, max_depth=7, learning_rate=0.05, min_samples_leaf=1: 0.6822

R^2 with n_estimators=100, max_depth=7, learning_rate=0.05, min_samples_leaf=2: 0.6875

R^2 with n_estimators=100, max_depth=7, learning_rate=0.05, min_samples_leaf=4: 0.6837

R^2 with n_estimators=100, max_depth=7, learning_rate=0.1, min_samples_leaf=1: 0.6822

R^2 with n_estimators=100, max_depth=7, learning_rate=0.1, min_samples_leaf=2: 0.6906

R^2 with n_estimators=100, max_depth=7, learning_rate=0.1, min_samples_leaf=4: 0.6855

R^2 with n_estimators=100, max_depth=10, learning_rate=0.01, min_samples_leaf=1: 0.5876

R^2 with n_estimators=100, max_depth=10, learning_rate=0.01, min_samples_leaf=2: 0.5878

R^2 with n_estimators=100, max_depth=10, learning_rate=0.01, min_samples_leaf=4: 0.5922

R^2 with n_estimators=100, max_depth=10, learning_rate=0.05, min_samples_leaf=1: 0.6765

R^2 with n_estimators=100, max_depth=10, learning_rate=0.05, min_samples_leaf=2: 0.6747

R^2 with n_estimators=100, max_depth=10, learning_rate=0.05, min_samples_leaf=4: 0.6859

R^2 with n_estimators=100, max_depth=10, learning_rate=0.1, min_samples_leaf=1: 0.6672

R^2 with n_estimators=100, max_depth=10, learning_rate=0.1, min_samples_leaf=2: 0.6669

R^2 with n_estimators=100, max_depth=10, learning_rate=0.1, min_samples_leaf=4: 0.6650

R^2 with n_estimators=110, max_depth=3, learning_rate=0.01, min_samples_leaf=1: 0.3666

R^2 with n_estimators=110, max_depth=3, learning_rate=0.01, min_samples_leaf=2: 0.3666

R^2 with n_estimators=110, max_depth=3, learning_rate=0.01, min_samples_leaf=4: 0.3667

R^2 with n_estimators=110, max_depth=3, learning_rate=0.05, min_samples_leaf=1: 0.5334

R^2 with n_estimators=110, max_depth=3, learning_rate=0.05, min_samples_leaf=2: 0.5320

R^2 with n_estimators=110, max_depth=3, learning_rate=0.05, min_samples_leaf=4: 0.5298

R^2 with n_estimators=110, max_depth=3, learning_rate=0.1, min_samples_leaf=1: 0.5788

R^2 with n_estimators=110, max_depth=3, learning_rate=0.1, min_samples_leaf=2: 0.5799

R^2 with n_estimators=110, max_depth=3, learning_rate=0.1, min_samples_leaf=4: 0.5820

R^2 with n_estimators=110, max_depth=5, learning_rate=0.01, min_samples_leaf=1: 0.4691

R^2 with n_estimators=110, max_depth=5, learning_rate=0.01, min_samples_leaf=2: 0.4700

R^2 with n_estimators=110, max_depth=5, learning_rate=0.01, min_samples_leaf=4: 0.4682

R^2 with n_estimators=110, max_depth=5, learning_rate=0.05, min_samples_leaf=1: 0.6306

R^2 with n_estimators=110, max_depth=5, learning_rate=0.05, min_samples_leaf=2: 0.6297

R^2 with n_estimators=110, max_depth=5, learning_rate=0.05, min_samples_leaf=4: 0.6319

R^2 with n_estimators=110, max_depth=5, learning_rate=0.1, min_samples_leaf=1: 0.6558

R^2 with n_estimators=110, max_depth=5, learning_rate=0.1, min_samples_leaf=2: 0.6659

R^2 with n_estimators=110, max_depth=5, learning_rate=0.1, min_samples_leaf=4: 0.6620

R^2 with n_estimators=110, max_depth=7, learning_rate=0.01, min_samples_leaf=1: 0.5415

R^2 with n_estimators=110, max_depth=7, learning_rate=0.01, min_samples_leaf=2: 0.5439

R^2 with n_estimators=110, max_depth=7, learning_rate=0.01, min_samples_leaf=4: 0.5416

R^2 with n_estimators=110, max_depth=7, learning_rate=0.05, min_samples_leaf=1: 0.6842

R^2 with n_estimators=110, max_depth=7, learning_rate=0.05, min_samples_leaf=2: 0.6891

R^2 with n_estimators=110, max_depth=7, learning_rate=0.05, min_samples_leaf=4: 0.6855

R^2 with n_estimators=110, max_depth=7, learning_rate=0.1, min_samples_leaf=1: 0.6831

R^2 with n_estimators=110, max_depth=7, learning_rate=0.1, min_samples_leaf=2: 0.6900

R^2 with n_estimators=110, max_depth=7, learning_rate=0.1, min_samples_leaf=4: 0.6868

R^2 with n_estimators=110, max_depth=10, learning_rate=0.01, min_samples_leaf=1: 0.6055

R^2 with n_estimators=110, max_depth=10, learning_rate=0.01, min_samples_leaf=2: 0.6054

R^2 with n_estimators=110, max_depth=10, learning_rate=0.01, min_samples_leaf=4: 0.6089

R^2 with n_estimators=110, max_depth=10, learning_rate=0.05, min_samples_leaf=1: 0.6753

R^2 with n_estimators=110, max_depth=10, learning_rate=0.05, min_samples_leaf=2: 0.6734

R^2 with n_estimators=110, max_depth=10, learning_rate=0.05, min_samples_leaf=4: 0.6848

R^2 with n_estimators=110, max_depth=10, learning_rate=0.1, min_samples_leaf=1: 0.6657

R^2 with n_estimators=110, max_depth=10, learning_rate=0.1, min_samples_leaf=2: 0.6664

R^2 with n_estimators=110, max_depth=10, learning_rate=0.1, min_samples_leaf=4: 0.6639

R^2 with n_estimators=150, max_depth=3, learning_rate=0.01, min_samples_leaf=1: 0.4093

R^2 with n_estimators=150, max_depth=3, learning_rate=0.01, min_samples_leaf=2: 0.4093

R^2 with n_estimators=150, max_depth=3, learning_rate=0.01, min_samples_leaf=4: 0.4092

R^2 with n_estimators=150, max_depth=3, learning_rate=0.05, min_samples_leaf=1: 0.5534

R^2 with n_estimators=150, max_depth=3, learning_rate=0.05, min_samples_leaf=2: 0.5502

R^2 with n_estimators=150, max_depth=3, learning_rate=0.05, min_samples_leaf=4: 0.5496

R^2 with n_estimators=150, max_depth=3, learning_rate=0.1, min_samples_leaf=1: 0.5960

R^2 with n_estimators=150, max_depth=3, learning_rate=0.1, min_samples_leaf=2: 0.5950

R^2 with n_estimators=150, max_depth=3, learning_rate=0.1, min_samples_leaf=4: 0.5970

R^2 with n_estimators=150, max_depth=5, learning_rate=0.01, min_samples_leaf=1: 0.5192

R^2 with n_estimators=150, max_depth=5, learning_rate=0.01, min_samples_leaf=2: 0.5206

R^2 with n_estimators=150, max_depth=5, learning_rate=0.01, min_samples_leaf=4: 0.5181

R^2 with n_estimators=150, max_depth=5, learning_rate=0.05, min_samples_leaf=1: 0.6425

R^2 with n_estimators=150, max_depth=5, learning_rate=0.05, min_samples_leaf=2: 0.6458

R^2 with n_estimators=150, max_depth=5, learning_rate=0.05, min_samples_leaf=4: 0.6464

R^2 with n_estimators=150, max_depth=5, learning_rate=0.1, min_samples_leaf=1: 0.6649

R^2 with n_estimators=150, max_depth=5, learning_rate=0.1, min_samples_leaf=2: 0.6744

R^2 with n_estimators=150, max_depth=5, learning_rate=0.1, min_samples_leaf=4: 0.6734

R^2 with n_estimators=150, max_depth=7, learning_rate=0.01, min_samples_leaf=1: 0.5942

R^2 with n_estimators=150, max_depth=7, learning_rate=0.01, min_samples_leaf=2: 0.5977

R^2 with n_estimators=150, max_depth=7, learning_rate=0.01, min_samples_leaf=4: 0.5953

R^2 with n_estimators=150, max_depth=7, learning_rate=0.05, min_samples_leaf=1: 0.6849

R^2 with n_estimators=150, max_depth=7, learning_rate=0.05, min_samples_leaf=2: 0.6929

R^2 with n_estimators=150, max_depth=7, learning_rate=0.05, min_samples_leaf=4: 0.6879

R^2 with n_estimators=150, max_depth=7, learning_rate=0.1, min_samples_leaf=1: 0.6829

R^2 with n_estimators=150, max_depth=7, learning_rate=0.1, min_samples_leaf=2: 0.6893

R^2 with n_estimators=150, max_depth=7, learning_rate=0.1, min_samples_leaf=4: 0.6876

R^2 with n_estimators=150, max_depth=10, learning_rate=0.01, min_samples_leaf=1: 0.6496

R^2 with n_estimators=150, max_depth=10, learning_rate=0.01, min_samples_leaf=2: 0.6503

R^2 with n_estimators=150, max_depth=10, learning_rate=0.01, min_samples_leaf=4: 0.6518

R^2 with n_estimators=150, max_depth=10, learning_rate=0.05, min_samples_leaf=1: 0.6731

R^2 with n_estimators=150, max_depth=10, learning_rate=0.05, min_samples_leaf=2: 0.6704

R^2 with n_estimators=150, max_depth=10, learning_rate=0.05, min_samples_leaf=4: 0.6814

R^2 with n_estimators=150, max_depth=10, learning_rate=0.1, min_samples_leaf=1: 0.6623

R^2 with n_estimators=150, max_depth=10, learning_rate=0.1, min_samples_leaf=2: 0.6621

R^2 with n_estimators=150, max_depth=10, learning_rate=0.1, min_samples_leaf=4: 0.6603

Best parameters found in the normalized dataset:
{'n_estimators': 150, 'max_depth': 7, 'learning_rate': 0.05, 'min_samples_leaf': 2}

X_train, X_test, y_train, y_test = train_test_split(X, y, test_size=0.15, random_state=42)
R² of the final model (normalized): 0.6621
RMSE of the final model (normalized): 0.5849

kf = KFold(n_splits=5, shuffle=True, random_state=42)
Mean R² in cross-validation: 0.7195280689752157

1.3 XGBoost

```
# Testing different parameter combinations for XGBoost on the normalized dataset
param_grid_normalized = {
    'n_estimators': [10, 50, 100, 110, 150],
    'max_depth': [3, 5, 7, 10],
    'learning_rate': [0.01, 0.05, 0.1],
    'min_child_weight': [1, 2, 4]
}
```

Testing different parameter combinations on the normalized dataset:
R² with n_estimators=10, max_depth=3, learning_rate=0.01, min_child_weight=1: 0.0660
R² with n_estimators=10, max_depth=3, learning_rate=0.01, min_child_weight=2: 0.0660
R² with n_estimators=10, max_depth=3, learning_rate=0.01, min_child_weight=4: 0.0660
R² with n_estimators=10, max_depth=3, learning_rate=0.05, min_child_weight=1: 0.2497
R² with n_estimators=10, max_depth=3, learning_rate=0.05, min_child_weight=2: 0.2497
R² with n_estimators=10, max_depth=3, learning_rate=0.05, min_child_weight=4: 0.2497
R² with n_estimators=10, max_depth=3, learning_rate=0.1, min_child_weight=1: 0.3589
R² with n_estimators=10, max_depth=3, learning_rate=0.1, min_child_weight=2: 0.3589
R² with n_estimators=10, max_depth=3, learning_rate=0.1, min_child_weight=4: 0.3589
R² with n_estimators=10, max_depth=5, learning_rate=0.01, min_child_weight=1: 0.0832
R² with n_estimators=10, max_depth=5, learning_rate=0.01, min_child_weight=2: 0.0832
R² with n_estimators=10, max_depth=5, learning_rate=0.01, min_child_weight=4: 0.0830
R² with n_estimators=10, max_depth=5, learning_rate=0.05, min_child_weight=1: 0.3195
R² with n_estimators=10, max_depth=5, learning_rate=0.05, min_child_weight=2: 0.3191
R² with n_estimators=10, max_depth=5, learning_rate=0.05, min_child_weight=4: 0.3185

R^2 with n_estimators=10, max_depth=5, learning_rate=0.1, min_child_weight=1: 0.4536

R^2 with n_estimators=10, max_depth=5, learning_rate=0.1, min_child_weight=2: 0.4521

R^2 with n_estimators=10, max_depth=5, learning_rate=0.1, min_child_weight=4: 0.4550

R^2 with n_estimators=10, max_depth=7, learning_rate=0.01, min_child_weight=1: 0.0972

R^2 with n_estimators=10, max_depth=7, learning_rate=0.01, min_child_weight=2: 0.0973

R^2 with n_estimators=10, max_depth=7, learning_rate=0.01, min_child_weight=4: 0.0967

R^2 with n_estimators=10, max_depth=7, learning_rate=0.05, min_child_weight=1: 0.3699

R^2 with n_estimators=10, max_depth=7, learning_rate=0.05, min_child_weight=2: 0.3687

R^2 with n_estimators=10, max_depth=7, learning_rate=0.05, min_child_weight=4: 0.3675

R^2 with n_estimators=10, max_depth=7, learning_rate=0.1, min_child_weight=1: 0.5249

R^2 with n_estimators=10, max_depth=7, learning_rate=0.1, min_child_weight=2: 0.5248

R^2 with n_estimators=10, max_depth=7, learning_rate=0.1, min_child_weight=4: 0.5254

R^2 with n_estimators=10, max_depth=10, learning_rate=0.01, min_child_weight=1: 0.1148

R^2 with n_estimators=10, max_depth=10, learning_rate=0.01, min_child_weight=2: 0.1153

R^2 with n_estimators=10, max_depth=10, learning_rate=0.01, min_child_weight=4: 0.1142

R^2 with n_estimators=10, max_depth=10, learning_rate=0.05, min_child_weight=1: 0.4257

R^2 with n_estimators=10, max_depth=10, learning_rate=0.05, min_child_weight=2: 0.4245

R^2 with n_estimators=10, max_depth=10, learning_rate=0.05, min_child_weight=4: 0.4231

R^2 with n_estimators=10, max_depth=10, learning_rate=0.1, min_child_weight=1: 0.5905

R^2 with n_estimators=10, max_depth=10, learning_rate=0.1, min_child_weight=2: 0.5930

R^2 with n_estimators=10, max_depth=10, learning_rate=0.1, min_child_weight=4: 0.5910

R^2 with n_estimators=50, max_depth=3, learning_rate=0.01, min_child_weight=1: 0.2453

R^2 with n_estimators=50, max_depth=3, learning_rate=0.01, min_child_weight=2: 0.2453

R^2 with n_estimators=50, max_depth=3, learning_rate=0.01, min_child_weight=4: 0.2453

R^2 with n_estimators=50, max_depth=3, learning_rate=0.05, min_child_weight=1: 0.4663

R^2 with n_estimators=50, max_depth=3, learning_rate=0.05, min_child_weight=2: 0.4664

R^2 with n_estimators=50, max_depth=3, learning_rate=0.05, min_child_weight=4: 0.4657

R^2 with n_estimators=50, max_depth=3, learning_rate=0.1, min_child_weight=1: 0.5271

R^2 with n_estimators=50, max_depth=3, learning_rate=0.1, min_child_weight=2: 0.5270

R^2 with n_estimators=50, max_depth=3, learning_rate=0.1, min_child_weight=4: 0.5270

R^2 with n_estimators=50, max_depth=5, learning_rate=0.01, min_child_weight=1: 0.3157

R^2 with n_estimators=50, max_depth=5, learning_rate=0.01, min_child_weight=2: 0.3156

R^2 with n_estimators=50, max_depth=5, learning_rate=0.01, min_child_weight=4: 0.3149

R^2 with n_estimators=50, max_depth=5, learning_rate=0.05, min_child_weight=1: 0.5766

R^2 with n_estimators=50, max_depth=5, learning_rate=0.05, min_child_weight=2: 0.5754

R^2 with n_estimators=50, max_depth=5, learning_rate=0.05, min_child_weight=4: 0.5757

R^2 with n_estimators=50, max_depth=5, learning_rate=0.1, min_child_weight=1: 0.6248

R^2 with n_estimators=50, max_depth=5, learning_rate=0.1, min_child_weight=2: 0.6214

R^2 with n_estimators=50, max_depth=5, learning_rate=0.1, min_child_weight=4: 0.6249

R^2 with n_estimators=50, max_depth=7, learning_rate=0.01, min_child_weight=1: 0.3644

R^2 with n_estimators=50, max_depth=7, learning_rate=0.01, min_child_weight=2: 0.3646

R^2 with n_estimators=50, max_depth=7, learning_rate=0.01, min_child_weight=4: 0.3627

R^2 with n_estimators=50, max_depth=7, learning_rate=0.05, min_child_weight=1: 0.6537

R^2 with n_estimators=50, max_depth=7, learning_rate=0.05, min_child_weight=2: 0.6557

R^2 with n_estimators=50, max_depth=7, learning_rate=0.05, min_child_weight=4: 0.6476

R^2 with n_estimators=50, max_depth=7, learning_rate=0.1, min_child_weight=1: 0.6829

R^2 with n_estimators=50, max_depth=7, learning_rate=0.1, min_child_weight=2: 0.6786

R^2 with n_estimators=50, max_depth=7, learning_rate=0.1, min_child_weight=4: 0.6810

R^2 with n_estimators=50, max_depth=10, learning_rate=0.01, min_child_weight=1: 0.4208

R^2 with n_estimators=50, max_depth=10, learning_rate=0.01, min_child_weight=2: 0.4214

R^2 with n_estimators=50, max_depth=10, learning_rate=0.01, min_child_weight=4: 0.4185

R^2 with n_estimators=50, max_depth=10, learning_rate=0.05, min_child_weight=1: 0.6878

R^2 with n_estimators=50, max_depth=10, learning_rate=0.05, min_child_weight=2: 0.6884

R^2 with n_estimators=50, max_depth=10, learning_rate=0.05, min_child_weight=4: 0.6899

R^2 with n_estimators=50, max_depth=10, learning_rate=0.1, min_child_weight=1: 0.6819

R^2 with n_estimators=50, max_depth=10, learning_rate=0.1, min_child_weight=2: 0.6866

R^2 with n_estimators=50, max_depth=10, learning_rate=0.1, min_child_weight=4: 0.6910

R^2 with n_estimators=100, max_depth=3, learning_rate=0.01, min_child_weight=1: 0.3510

R^2 with n_estimators=100, max_depth=3, learning_rate=0.01, min_child_weight=2: 0.3510

R^2 with n_estimators=100, max_depth=3, learning_rate=0.01, min_child_weight=4: 0.3510

R^2 with n_estimators=100, max_depth=3, learning_rate=0.05, min_child_weight=1: 0.5254

R^2 with n_estimators=100, max_depth=3, learning_rate=0.05, min_child_weight=2: 0.5255

R^2 with n_estimators=100, max_depth=3, learning_rate=0.05, min_child_weight=4: 0.5255

R^2 with n_estimators=100, max_depth=3, learning_rate=0.1, min_child_weight=1: 0.5743

R^2 with n_estimators=100, max_depth=3, learning_rate=0.1, min_child_weight=2: 0.5782

R^2 with n_estimators=100, max_depth=3, learning_rate=0.1, min_child_weight=4: 0.5759

R^2 with n_estimators=100, max_depth=5, learning_rate=0.01, min_child_weight=1: 0.4489

R^2 with n_estimators=100, max_depth=5, learning_rate=0.01, min_child_weight=2: 0.4490

R^2 with n_estimators=100, max_depth=5, learning_rate=0.01, min_child_weight=4: 0.4488

R^2 with n_estimators=100, max_depth=5, learning_rate=0.05, min_child_weight=1: 0.6277

R^2 with n_estimators=100, max_depth=5, learning_rate=0.05, min_child_weight=2: 0.6283

R^2 with n_estimators=100, max_depth=5, learning_rate=0.05, min_child_weight=4: 0.6273

R^2 with n_estimators=100, max_depth=5, learning_rate=0.1, min_child_weight=1: 0.6544

R^2 with n_estimators=100, max_depth=5, learning_rate=0.1, min_child_weight=2: 0.6528

R^2 with n_estimators=100, max_depth=5, learning_rate=0.1, min_child_weight=4: 0.6589

R^2 with n_estimators=100, max_depth=7, learning_rate=0.01, min_child_weight=1: 0.5215

R^2 with n_estimators=100, max_depth=7, learning_rate=0.01, min_child_weight=2: 0.5211

R^2 with n_estimators=100, max_depth=7, learning_rate=0.01, min_child_weight=4: 0.5176

R^2 with n_estimators=100, max_depth=7, learning_rate=0.05, min_child_weight=1: 0.6867

R^2 with n_estimators=100, max_depth=7, learning_rate=0.05, min_child_weight=2: 0.6867

R^2 with n_estimators=100, max_depth=7, learning_rate=0.05, min_child_weight=4: 0.6801

R^2 with n_estimators=100, max_depth=7, learning_rate=0.1, min_child_weight=1: 0.6914

R^2 with n_estimators=100, max_depth=7, learning_rate=0.1, min_child_weight=2: 0.6888

R^2 with n_estimators=100, max_depth=7, learning_rate=0.1, min_child_weight=4: 0.6912

R^2 with n_estimators=100, max_depth=10, learning_rate=0.01, min_child_weight=1: 0.5827

R^2 with n_estimators=100, max_depth=10, learning_rate=0.01, min_child_weight=2: 0.5851

R^2 with n_estimators=100, max_depth=10, learning_rate=0.01, min_child_weight=4: 0.5835

R^2 with n_estimators=100, max_depth=10, learning_rate=0.05, min_child_weight=1: 0.6882

R^2 with n_estimators=100, max_depth=10, learning_rate=0.05, min_child_weight=2: 0.6893

R^2 with n_estimators=100, max_depth=10, learning_rate=0.05, min_child_weight=4: 0.6880

R^2 with n_estimators=100, max_depth=10, learning_rate=0.1, min_child_weight=1: 0.6726

R^2 with n_estimators=100, max_depth=10, learning_rate=0.1, min_child_weight=2: 0.6762

R^2 with n_estimators=100, max_depth=10, learning_rate=0.1, min_child_weight=4: 0.6818

R^2 with n_estimators=110, max_depth=3, learning_rate=0.01, min_child_weight=1: 0.3663

R^2 with n_estimators=110, max_depth=3, learning_rate=0.01, min_child_weight=2: 0.3663

R^2 with n_estimators=110, max_depth=3, learning_rate=0.01, min_child_weight=4: 0.3663

R^2 with n_estimators=110, max_depth=3, learning_rate=0.05, min_child_weight=1: 0.5339

R^2 with n_estimators=110, max_depth=3, learning_rate=0.05, min_child_weight=2: 0.5321

R^2 with n_estimators=110, max_depth=3, learning_rate=0.05, min_child_weight=4: 0.5329

R^2 with n_estimators=110, max_depth=3, learning_rate=0.1, min_child_weight=1: 0.5809

R^2 with n_estimators=110, max_depth=3, learning_rate=0.1, min_child_weight=2: 0.5836

R^2 with n_estimators=110, max_depth=3, learning_rate=0.1, min_child_weight=4: 0.5833

R^2 with n_estimators=110, max_depth=5, learning_rate=0.01, min_child_weight=1: 0.4658

R^2 with n_estimators=110, max_depth=5, learning_rate=0.01, min_child_weight=2: 0.4655

R^2 with n_estimators=110, max_depth=5, learning_rate=0.01, min_child_weight=4: 0.4655

R^2 with n_estimators=110, max_depth=5, learning_rate=0.05, min_child_weight=1: 0.6320

R^2 with n_estimators=110, max_depth=5, learning_rate=0.05, min_child_weight=2: 0.6328

R^2 with n_estimators=110, max_depth=5, learning_rate=0.05, min_child_weight=4: 0.6332

R^2 with n_estimators=110, max_depth=5, learning_rate=0.1, min_child_weight=1: 0.6579

R^2 with n_estimators=110, max_depth=5, learning_rate=0.1, min_child_weight=2: 0.6568

R^2 with n_estimators=110, max_depth=5, learning_rate=0.1, min_child_weight=4: 0.6631

R^2 with n_estimators=110, max_depth=7, learning_rate=0.01, min_child_weight=1: 0.5407

R^2 with n_estimators=110, max_depth=7, learning_rate=0.01, min_child_weight=2: 0.5400

R^2 with n_estimators=110, max_depth=7, learning_rate=0.01, min_child_weight=4: 0.5361

R^2 with n_estimators=110, max_depth=7, learning_rate=0.05, min_child_weight=1: 0.6887

R^2 with n_estimators=110, max_depth=7, learning_rate=0.05, min_child_weight=2: 0.6893

R^2 with n_estimators=110, max_depth=7, learning_rate=0.05, min_child_weight=4: 0.6823

R^2 with n_estimators=110, max_depth=7, learning_rate=0.1, min_child_weight=1: 0.6928

R^2 with n_estimators=110, max_depth=7, learning_rate=0.1, min_child_weight=2: 0.6896

R^2 with n_estimators=110, max_depth=7, learning_rate=0.1, min_child_weight=4: 0.6922

R^2 with n_estimators=110, max_depth=10, learning_rate=0.01, min_child_weight=1: 0.6015

R^2 with n_estimators=110, max_depth=10, learning_rate=0.01, min_child_weight=2: 0.6038

R^2 with n_estimators=110, max_depth=10, learning_rate=0.01, min_child_weight=4: 0.6008

R^2 with n_estimators=110, max_depth=10, learning_rate=0.05, min_child_weight=1: 0.6868

R^2 with n_estimators=110, max_depth=10, learning_rate=0.05, min_child_weight=2: 0.6880

R^2 with n_estimators=110, max_depth=10, learning_rate=0.05, min_child_weight=4: 0.6867

R^2 with n_estimators=110, max_depth=10, learning_rate=0.1, min_child_weight=1: 0.6719

R^2 with n_estimators=110, max_depth=10, learning_rate=0.1, min_child_weight=2: 0.6745

R^2 with n_estimators=110, max_depth=10, learning_rate=0.1, min_child_weight=4: 0.6800

R^2 with n_estimators=150, max_depth=3, learning_rate=0.01, min_child_weight=1: 0.4089

R^2 with n_estimators=150, max_depth=3, learning_rate=0.01, min_child_weight=2: 0.4089

R^2 with n_estimators=150, max_depth=3, learning_rate=0.01, min_child_weight=4: 0.4089

R^2 with n_estimators=150, max_depth=3, learning_rate=0.05, min_child_weight=1: 0.5570

R^2 with n_estimators=150, max_depth=3, learning_rate=0.05, min_child_weight=2: 0.5563

R^2 with n_estimators=150, max_depth=3, learning_rate=0.05, min_child_weight=4: 0.5554

R^2 with n_estimators=150, max_depth=3, learning_rate=0.1, min_child_weight=1: 0.6000

R^2 with n_estimators=150, max_depth=3, learning_rate=0.1, min_child_weight=2: 0.6005

R^2 with n_estimators=150, max_depth=3, learning_rate=0.1, min_child_weight=4: 0.5999

R^2 with n_estimators=150, max_depth=5, learning_rate=0.01, min_child_weight=1: 0.5144

R^2 with n_estimators=150, max_depth=5, learning_rate=0.01, min_child_weight=2: 0.5142

R^2 with n_estimators=150, max_depth=5, learning_rate=0.01, min_child_weight=4: 0.5133

R^2 with n_estimators=150, max_depth=5, learning_rate=0.05, min_child_weight=1: 0.6471

R^2 with n_estimators=150, max_depth=5, learning_rate=0.05, min_child_weight=2: 0.6474

R^2 with n_estimators=150, max_depth=5, learning_rate=0.05, min_child_weight=4: 0.6475

R^2 with n_estimators=150, max_depth=5, learning_rate=0.1, min_child_weight=1: 0.6710

R^2 with n_estimators=150, max_depth=5, learning_rate=0.1, min_child_weight=2: 0.6712

R^2 with n_estimators=150, max_depth=5, learning_rate=0.1, min_child_weight=4: 0.6740

R^2 with n_estimators=150, max_depth=7, learning_rate=0.01, min_child_weight=1: 0.5918

R^2 with n_estimators=150, max_depth=7, learning_rate=0.01, min_child_weight=2: 0.5948
R^2 with n_estimators=150, max_depth=7, learning_rate=0.01, min_child_weight=4: 0.5875
R^2 with n_estimators=150, max_depth=7, learning_rate=0.05, min_child_weight=1: 0.6910
R^2 with n_estimators=150, max_depth=7, learning_rate=0.05, min_child_weight=2: 0.6945
R^2 with n_estimators=150, max_depth=7, learning_rate=0.05, min_child_weight=4: 0.6877
R^2 with n_estimators=150, max_depth=7, learning_rate=0.1, min_child_weight=1: 0.6919
R^2 with n_estimators=150, max_depth=7, learning_rate=0.1, min_child_weight=2: 0.6879
R^2 with n_estimators=150, max_depth=7, learning_rate=0.1, min_child_weight=4: 0.6921
R^2 with n_estimators=150, max_depth=10, learning_rate=0.01, min_child_weight=1: 0.6506
R^2 with n_estimators=150, max_depth=10, learning_rate=0.01, min_child_weight=2: 0.6527
R^2 with n_estimators=150, max_depth=10, learning_rate=0.01, min_child_weight=4: 0.6507
R^2 with n_estimators=150, max_depth=10, learning_rate=0.05, min_child_weight=1: 0.6834
R^2 with n_estimators=150, max_depth=10, learning_rate=0.05, min_child_weight=2: 0.6839
R^2 with n_estimators=150, max_depth=10, learning_rate=0.05, min_child_weight=4: 0.6833
R^2 with n_estimators=150, max_depth=10, learning_rate=0.1, min_child_weight=1: 0.6689
R^2 with n_estimators=150, max_depth=10, learning_rate=0.1, min_child_weight=2: 0.6707
R^2 with n_estimators=150, max_depth=10, learning_rate=0.1, min_child_weight=4: 0.6754

Best parameters found in the normalized dataset:
{'n_estimators': 150, 'max_depth': 7, 'learning_rate': 0.05, 'min_child_weight': 2}

X_train, X_test, y_train, y_test = train_test_split(X, y, test_size=0.15, random_state=42)
R^2 of the final model (normalized): 0.5734
RMSE of the final model (normalized): 0.6572

kf = KFold(n_splits=5, shuffle=True, random_state=42)
Mean R^2 in cross-validation: 0.7211107786093788

1.4 LightGBM

Testing different parameter combinations for LightGBM on the normalized dataset:
param_grid_normalized = {
'n_estimators': [10, 50, 100, 110, 150],

```
    'max_depth': [3, 5, 7, 10],
    'learning_rate': [0.01, 0.05, 0.1],
    'min_child_samples': [1, 5, 10]
}
```

R^2 with n_estimators=10, max_depth=3, learning_rate=0.01, min_child_samples=1: 0.0660
R^2 with n_estimators=10, max_depth=3, learning_rate=0.01, min_child_samples=5: 0.0660
R^2 with n_estimators=10, max_depth=3, learning_rate=0.01, min_child_samples=10: 0.0660
R^2 with n_estimators=10, max_depth=3, learning_rate=0.05, min_child_samples=1: 0.2499
R^2 with n_estimators=10, max_depth=3, learning_rate=0.05, min_child_samples=5: 0.2499
R^2 with n_estimators=10, max_depth=3, learning_rate=0.05, min_child_samples=10: 0.2492
R^2 with n_estimators=10, max_depth=3, learning_rate=0.1, min_child_samples=1: 0.3593
R^2 with n_estimators=10, max_depth=3, learning_rate=0.1, min_child_samples=5: 0.3591
R^2 with n_estimators=10, max_depth=3, learning_rate=0.1, min_child_samples=10: 0.3606
R^2 with n_estimators=10, max_depth=5, learning_rate=0.01, min_child_samples=1: 0.0838
R^2 with n_estimators=10, max_depth=5, learning_rate=0.01, min_child_samples=5: 0.0834
R^2 with n_estimators=10, max_depth=5, learning_rate=0.01, min_child_samples=10: 0.0842
R^2 with n_estimators=10, max_depth=5, learning_rate=0.05, min_child_samples=1: 0.3212
R^2 with n_estimators=10, max_depth=5, learning_rate=0.05, min_child_samples=5: 0.3199
R^2 with n_estimators=10, max_depth=5, learning_rate=0.05, min_child_samples=10: 0.3179
R^2 with n_estimators=10, max_depth=5, learning_rate=0.1, min_child_samples=1: 0.4581
R^2 with n_estimators=10, max_depth=5, learning_rate=0.1, min_child_samples=5: 0.4562
R^2 with n_estimators=10, max_depth=5, learning_rate=0.1, min_child_samples=10: 0.4553
R^2 with n_estimators=10, max_depth=7, learning_rate=0.01, min_child_samples=1: 0.0892
R^2 with n_estimators=10, max_depth=7, learning_rate=0.01, min_child_samples=5: 0.0892
R^2 with n_estimators=10, max_depth=7, learning_rate=0.01, min_child_samples=10: 0.0892
R^2 with n_estimators=10, max_depth=7, learning_rate=0.05, min_child_samples=1: 0.3327
R^2 with n_estimators=10, max_depth=7, learning_rate=0.05, min_child_samples=5: 0.3324
R^2 with n_estimators=10, max_depth=7, learning_rate=0.05, min_child_samples=10: 0.3331
R^2 with n_estimators=10, max_depth=7, learning_rate=0.1, min_child_samples=1: 0.4744
R^2 with n_estimators=10, max_depth=7, learning_rate=0.1, min_child_samples=5: 0.4743
R^2 with n_estimators=10, max_depth=7, learning_rate=0.1, min_child_samples=10: 0.4748
R^2 with n_estimators=10, max_depth=10, learning_rate=0.01, min_child_samples=1: 0.0891
R^2 with n_estimators=10, max_depth=10, learning_rate=0.01, min_child_samples=5: 0.0891
R^2 with n_estimators=10, max_depth=10, learning_rate=0.01, min_child_samples=10: 0.0892
R^2 with n_estimators=10, max_depth=10, learning_rate=0.05, min_child_samples=1: 0.3312
R^2 with n_estimators=10, max_depth=10, learning_rate=0.05, min_child_samples=5: 0.3312

R^2 with n_estimators=10, max_depth=10, learning_rate=0.05, min_child_samples=10: 0.3331

R^2 with n_estimators=10, max_depth=10, learning_rate=0.1, min_child_samples=1: 0.4711

R^2 with n_estimators=10, max_depth=10, learning_rate=0.1, min_child_samples=5: 0.4711

R^2 with n_estimators=10, max_depth=10, learning_rate=0.1, min_child_samples=10: 0.4734

R^2 with n_estimators=50, max_depth=3, learning_rate=0.01, min_child_samples=1: 0.2457

R^2 with n_estimators=50, max_depth=3, learning_rate=0.01, min_child_samples=5: 0.2457

R^2 with n_estimators=50, max_depth=3, learning_rate=0.01, min_child_samples=10: 0.2454

R^2 with n_estimators=50, max_depth=3, learning_rate=0.05, min_child_samples=1: 0.4674

R^2 with n_estimators=50, max_depth=3, learning_rate=0.05, min_child_samples=5: 0.4665

R^2 with n_estimators=50, max_depth=3, learning_rate=0.05, min_child_samples=10: 0.4639

R^2 with n_estimators=50, max_depth=3, learning_rate=0.1, min_child_samples=1: 0.5295

R^2 with n_estimators=50, max_depth=3, learning_rate=0.1, min_child_samples=5: 0.5282

R^2 with n_estimators=50, max_depth=3, learning_rate=0.1, min_child_samples=10: 0.5253

R^2 with n_estimators=50, max_depth=5, learning_rate=0.01, min_child_samples=1: 0.3160

R^2 with n_estimators=50, max_depth=5, learning_rate=0.01, min_child_samples=5: 0.3153

R^2 with n_estimators=50, max_depth=5, learning_rate=0.01, min_child_samples=10: 0.3147

R^2 with n_estimators=50, max_depth=5, learning_rate=0.05, min_child_samples=1: 0.5814

R^2 with n_estimators=50, max_depth=5, learning_rate=0.05, min_child_samples=5: 0.5802

R^2 with n_estimators=50, max_depth=5, learning_rate=0.05, min_child_samples=10: 0.5737

R^2 with n_estimators=50, max_depth=5, learning_rate=0.1, min_child_samples=1: 0.6287

R^2 with n_estimators=50, max_depth=5, learning_rate=0.1, min_child_samples=5: 0.6197

R^2 with n_estimators=50, max_depth=5, learning_rate=0.1, min_child_samples=10: 0.6234

R^2 with n_estimators=50, max_depth=7, learning_rate=0.01, min_child_samples=1: 0.3296

R^2 with n_estimators=50, max_depth=7, learning_rate=0.01, min_child_samples=5: 0.3297

R^2 with n_estimators=50, max_depth=7, learning_rate=0.01, min_child_samples=10: 0.3291

R^2 with n_estimators=50, max_depth=7, learning_rate=0.05, min_child_samples=1: 0.5963

R^2 with n_estimators=50, max_depth=7, learning_rate=0.05, min_child_samples=5: 0.5949

R^2 with n_estimators=50, max_depth=7, learning_rate=0.05, min_child_samples=10: 0.5938

R^2 with n_estimators=50, max_depth=7, learning_rate=0.1, min_child_samples=1: 0.6410

R^2 with n_estimators=50, max_depth=7, learning_rate=0.1, min_child_samples=5: 0.6409

R^2 with n_estimators=50, max_depth=7, learning_rate=0.1, min_child_samples=10: 0.6398

R^2 with n_estimators=50, max_depth=10, learning_rate=0.01, min_child_samples=1: 0.3275

R^2 with n_estimators=50, max_depth=10, learning_rate=0.01, min_child_samples=5: 0.3272

R^2 with n_estimators=50, max_depth=10, learning_rate=0.01, min_child_samples=10: 0.3286

R^2 with n_estimators=50, max_depth=10, learning_rate=0.05, min_child_samples=1: 0.5944

R^2 with n_estimators=50, max_depth=10, learning_rate=0.05, min_child_samples=5: 0.5928

R^2 with n_estimators=50, max_depth=10, learning_rate=0.05, min_child_samples=10: 0.5924

R^2 with n_estimators=50, max_depth=10, learning_rate=0.1, min_child_samples=1: 0.6384

R^2 with n_estimators=50, max_depth=10, learning_rate=0.1, min_child_samples=5: 0.6398

R^2 with n_estimators=50, max_depth=10, learning_rate=0.1, min_child_samples=10: 0.6420

R^2 with n_estimators=100, max_depth=3, learning_rate=0.01, min_child_samples=1: 0.3521

R^2 with n_estimators=100, max_depth=3, learning_rate=0.01, min_child_samples=5: 0.3520

R^2 with n_estimators=100, max_depth=3, learning_rate=0.01, min_child_samples=10: 0.3510

R^2 with n_estimators=100, max_depth=3, learning_rate=0.05, min_child_samples=1: 0.5315

R^2 with n_estimators=100, max_depth=3, learning_rate=0.05, min_child_samples=5: 0.5277

R^2 with n_estimators=100, max_depth=3, learning_rate=0.05, min_child_samples=10: 0.5266

R^2 with n_estimators=100, max_depth=3, learning_rate=0.1, min_child_samples=1: 0.5740

R^2 with n_estimators=100, max_depth=3, learning_rate=0.1, min_child_samples=5: 0.5749

R^2 with n_estimators=100, max_depth=3, learning_rate=0.1, min_child_samples=10: 0.5671

R^2 with n_estimators=100, max_depth=5, learning_rate=0.01, min_child_samples=1: 0.4502

R^2 with n_estimators=100, max_depth=5, learning_rate=0.01, min_child_samples=5: 0.4501

R^2 with n_estimators=100, max_depth=5, learning_rate=0.01, min_child_samples=10: 0.4484

R^2 with n_estimators=100, max_depth=5, learning_rate=0.05, min_child_samples=1: 0.6267

R^2 with n_estimators=100, max_depth=5, learning_rate=0.05, min_child_samples=5: 0.6297

R^2 with n_estimators=100, max_depth=5, learning_rate=0.05, min_child_samples=10: 0.6239

R^2 with n_estimators=100, max_depth=5, learning_rate=0.1, min_child_samples=1: 0.6543

R^2 with n_estimators=100, max_depth=5, learning_rate=0.1, min_child_samples=5: 0.6574

R^2 with n_estimators=100, max_depth=5, learning_rate=0.1, min_child_samples=10: 0.6597

R^2 with n_estimators=100, max_depth=7, learning_rate=0.01, min_child_samples=1: 0.4674

R^2 with n_estimators=100, max_depth=7, learning_rate=0.01, min_child_samples=5: 0.4670

R^2 with n_estimators=100, max_depth=7, learning_rate=0.01, min_child_samples=10: 0.4660

R^2 with n_estimators=100, max_depth=7, learning_rate=0.05, min_child_samples=1: 0.6488

R^2 with n_estimators=100, max_depth=7, learning_rate=0.05, min_child_samples=5: 0.6456

R^2 with n_estimators=100, max_depth=7, learning_rate=0.05, min_child_samples=10: 0.6487

R^2 with n_estimators=100, max_depth=7, learning_rate=0.1, min_child_samples=1: 0.6718

R^2 with n_estimators=100, max_depth=7, learning_rate=0.1, min_child_samples=5: 0.6784

R^2 with n_estimators=100, max_depth=7, learning_rate=0.1, min_child_samples=10: 0.6715

R^2 with n_estimators=100, max_depth=10, learning_rate=0.01, min_child_samples=1: 0.4653

R^2 with n_estimators=100, max_depth=10, learning_rate=0.01, min_child_samples=5: 0.4655

R^2 with n_estimators=100, max_depth=10, learning_rate=0.01, min_child_samples=10: 0.4659

R^2 with n_estimators=100, max_depth=10, learning_rate=0.05, min_child_samples=1: 0.6474

R^2 with n_estimators=100, max_depth=10, learning_rate=0.05, min_child_samples=5: 0.6429

R^2 with n_estimators=100, max_depth=10, learning_rate=0.05, min_child_samples=10: 0.6455

R^2 with n_estimators=100, max_depth=10, learning_rate=0.1, min_child_samples=1: 0.6698

R^2 with n_estimators=100, max_depth=10, learning_rate=0.1, min_child_samples=5: 0.6728

R^2 with n_estimators=100, max_depth=10, learning_rate=0.1, min_child_samples=10: 0.6704

R^2 with n_estimators=110, max_depth=3, learning_rate=0.01, min_child_samples=1: 0.3670

R^2 with n_estimators=110, max_depth=3, learning_rate=0.01, min_child_samples=5: 0.3669

R^2 with n_estimators=110, max_depth=3, learning_rate=0.01, min_child_samples=10: 0.3660

R^2 with n_estimators=110, max_depth=3, learning_rate=0.05, min_child_samples=1: 0.5385

R^2 with n_estimators=110, max_depth=3, learning_rate=0.05, min_child_samples=5: 0.5358

R^2 with n_estimators=110, max_depth=3, learning_rate=0.05, min_child_samples=10: 0.5335

R^2 with n_estimators=110, max_depth=3, learning_rate=0.1, min_child_samples=1: 0.5808

R^2 with n_estimators=110, max_depth=3, learning_rate=0.1, min_child_samples=5: 0.5821

R^2 with n_estimators=110, max_depth=3, learning_rate=0.1, min_child_samples=10: 0.5734

R^2 with n_estimators=110, max_depth=5, learning_rate=0.01, min_child_samples=1: 0.4676

R^2 with n_estimators=110, max_depth=5, learning_rate=0.01, min_child_samples=5: 0.4675

R^2 with n_estimators=110, max_depth=5, learning_rate=0.01, min_child_samples=10: 0.4652

R^2 with n_estimators=110, max_depth=5, learning_rate=0.05, min_child_samples=1: 0.6322

R^2 with n_estimators=110, max_depth=5, learning_rate=0.05, min_child_samples=5: 0.6350

R^2 with n_estimators=110, max_depth=5, learning_rate=0.05, min_child_samples=10: 0.6293

R^2 with n_estimators=110, max_depth=5, learning_rate=0.1, min_child_samples=1: 0.6595

R^2 with n_estimators=110, max_depth=5, learning_rate=0.1, min_child_samples=5: 0.6604

R^2 with n_estimators=110, max_depth=5, learning_rate=0.1, min_child_samples=10: 0.6630

R^2 with n_estimators=110, max_depth=7, learning_rate=0.01, min_child_samples=1: 0.4853

R^2 with n_estimators=110, max_depth=7, learning_rate=0.01, min_child_samples=5: 0.4847

R^2 with n_estimators=110, max_depth=7, learning_rate=0.01, min_child_samples=10: 0.4833

R^2 with n_estimators=110, max_depth=7, learning_rate=0.05, min_child_samples=1: 0.6531

R^2 with n_estimators=110, max_depth=7, learning_rate=0.05, min_child_samples=5: 0.6495

R^2 with n_estimators=110, max_depth=7, learning_rate=0.05, min_child_samples=10: 0.6537

R^2 with n_estimators=110, max_depth=7, learning_rate=0.1, min_child_samples=1: 0.6749

R^2 with n_estimators=110, max_depth=7, learning_rate=0.1, min_child_samples=5: 0.6828

R^2 with n_estimators=110, max_depth=7, learning_rate=0.1, min_child_samples=10: 0.6749

R^2 with n_estimators=110, max_depth=10, learning_rate=0.01, min_child_samples=1: 0.4829

R^2 with n_estimators=110, max_depth=10, learning_rate=0.01, min_child_samples=5: 0.4831

R^2 with n_estimators=110, max_depth=10, learning_rate=0.01, min_child_samples=10: 0.4831

R^2 with n_estimators=110, max_depth=10, learning_rate=0.05, min_child_samples=1: 0.6521

R^2 with n_estimators=110, max_depth=10, learning_rate=0.05, min_child_samples=5: 0.6476

R^2 with n_estimators=110, max_depth=10, learning_rate=0.05, min_child_samples=10: 0.6495

R^2 with n_estimators=110, max_depth=10, learning_rate=0.1, min_child_samples=1: 0.6746

R^2 with n_estimators=110, max_depth=10, learning_rate=0.1, min_child_samples=5: 0.6768

R^2 with n_estimators=110, max_depth=10, learning_rate=0.1, min_child_samples=10: 0.6737
R^2 with n_estimators=150, max_depth=3, learning_rate=0.01, min_child_samples=1: 0.4099
R^2 with n_estimators=150, max_depth=3, learning_rate=0.01, min_child_samples=5: 0.4099
R^2 with n_estimators=150, max_depth=3, learning_rate=0.01, min_child_samples=10: 0.4087
R^2 with n_estimators=150, max_depth=3, learning_rate=0.05, min_child_samples=1: 0.5572
R^2 with n_estimators=150, max_depth=3, learning_rate=0.05, min_child_samples=5: 0.5559
R^2 with n_estimators=150, max_depth=3, learning_rate=0.05, min_child_samples=10: 0.5525
R^2 with n_estimators=150, max_depth=3, learning_rate=0.1, min_child_samples=1: 0.5970
R^2 with n_estimators=150, max_depth=3, learning_rate=0.1, min_child_samples=5: 0.6022
R^2 with n_estimators=150, max_depth=3, learning_rate=0.1, min_child_samples=10: 0.5955
R^2 with n_estimators=150, max_depth=5, learning_rate=0.01, min_child_samples=1: 0.5188
R^2 with n_estimators=150, max_depth=5, learning_rate=0.01, min_child_samples=5: 0.5180
R^2 with n_estimators=150, max_depth=5, learning_rate=0.01, min_child_samples=10: 0.5153
R^2 with n_estimators=150, max_depth=5, learning_rate=0.05, min_child_samples=1: 0.6478
R^2 with n_estimators=150, max_depth=5, learning_rate=0.05, min_child_samples=5: 0.6486
R^2 with n_estimators=150, max_depth=5, learning_rate=0.05, min_child_samples=10: 0.6446
R^2 with n_estimators=150, max_depth=5, learning_rate=0.1, min_child_samples=1: 0.6728
R^2 with n_estimators=150, max_depth=5, learning_rate=0.1, min_child_samples=5: 0.6715
R^2 with n_estimators=150, max_depth=5, learning_rate=0.1, min_child_samples=10: 0.6741
R^2 with n_estimators=150, max_depth=7, learning_rate=0.01, min_child_samples=1: 0.5347
R^2 with n_estimators=150, max_depth=7, learning_rate=0.01, min_child_samples=5: 0.5343
R^2 with n_estimators=150, max_depth=7, learning_rate=0.01, min_child_samples=10: 0.5319
R^2 with n_estimators=150, max_depth=7, learning_rate=0.05, min_child_samples=1: 0.6667
R^2 with n_estimators=150, max_depth=7, learning_rate=0.05, min_child_samples=5: 0.6639
R^2 with n_estimators=150, max_depth=7, learning_rate=0.05, min_child_samples=10: 0.6659
R^2 with n_estimators=150, max_depth=7, learning_rate=0.1, min_child_samples=1: 0.6809
R^2 with n_estimators=150, max_depth=7, learning_rate=0.1, min_child_samples=5: 0.6896
R^2 with n_estimators=150, max_depth=7, learning_rate=0.1, min_child_samples=10: 0.6830
R^2 with n_estimators=150, max_depth=10, learning_rate=0.01, min_child_samples=1: 0.5331
R^2 with n_estimators=150, max_depth=10, learning_rate=0.01, min_child_samples=5: 0.5339
R^2 with n_estimators=150, max_depth=10, learning_rate=0.01, min_child_samples=10: 0.5320
R^2 with n_estimators=150, max_depth=10, learning_rate=0.05, min_child_samples=1: 0.6663
R^2 with n_estimators=150, max_depth=10, learning_rate=0.05, min_child_samples=5: 0.6639
R^2 with n_estimators=150, max_depth=10, learning_rate=0.05, min_child_samples=10: 0.6642
R^2 with n_estimators=150, max_depth=10, learning_rate=0.1, min_child_samples=1: 0.6802
R^2 with n_estimators=150, max_depth=10, learning_rate=0.1, min_child_samples=5: 0.6820
R^2 with n_estimators=150, max_depth=10, learning_rate=0.1, min_child_samples=10: 0.6813

Best parameters found in the normalized dataset:

```
{'n_estimators': 150, 'max_depth': 7, 'learning_rate': 0.1, 'min_child_samples': 5}
```

```
X_train, X_test, y_train, y_test = train_test_split(X, y, test_size=0.15, random_state=42)
```

Final model R^2 (normalized): 0.6896

Final model RMSE (normalized): 0.5605

```
kf = KFold(n_splits=5, shuffle=True, random_state=42)
```

Average R^2 in cross-validation: 0.7260621674316756

2 Activity Cliffs - Test

Obtained validation after removing activity cliffs:

```
X_train, X_test, y_train, y_test = train_test_split(X, y, test_size=0.05, random_state=42)
#Y-scrambling test
from sklearn.utils import shuffle

n_iter = 100
r2_scores_scramble = []

for i in range(n_iter):
y_scrambled = shuffle(y, random_state=i)
rf_scramble = RandomForestRegressor(
n_estimators=100, max_depth=30, min_samples_leaf=1,
max_features='sqrt', random_state=42
)
rf_scramble.fit(X_train_normalized, y_scrambled.iloc[y_train.index])
r2_scramble = rf_scramble.score(X_test_normalized, y_test)
r2_scores_scramble.append(r2_scramble)

print(f"Mean R² Y-scrambled: {np.mean(r2_scores_scramble):.4f} ± {np.std(r2_scores_scramble):.4f}")
```

Mean R^2 Y-scrambled: -0.2100 ± 0.0590

Final model R^2 (normalized, train): 0.9632

Final model R^2 (normalized, test): 0.8207

Final model RMSE (normalized, train): 0.2050

Final model RMSE (normalized, test): 0.4652

Cross-validation results (mean metrics):

Mean MAE: 0.3548

Mean RMSE: 0.4858

Mean R^2: 0.7934

Mean MPD: 6.6355%

Mean MGD: 4.7763%

Standard deviation of MAE: 0.0091

Standard deviation of RMSE: 0.0132

Standard deviation of R^2: 0.0093

Standard deviation of MPD: 0.1943%

Standard deviation of MGD: 0.1828%

Number of molecules involved in activity cliffs: 1513

3 Examples of decision trees used according to the selected RF parameters*

* (n_estimators: 100; max_depth: 30; min_samples_leaf: 1; max_features: sqrt)

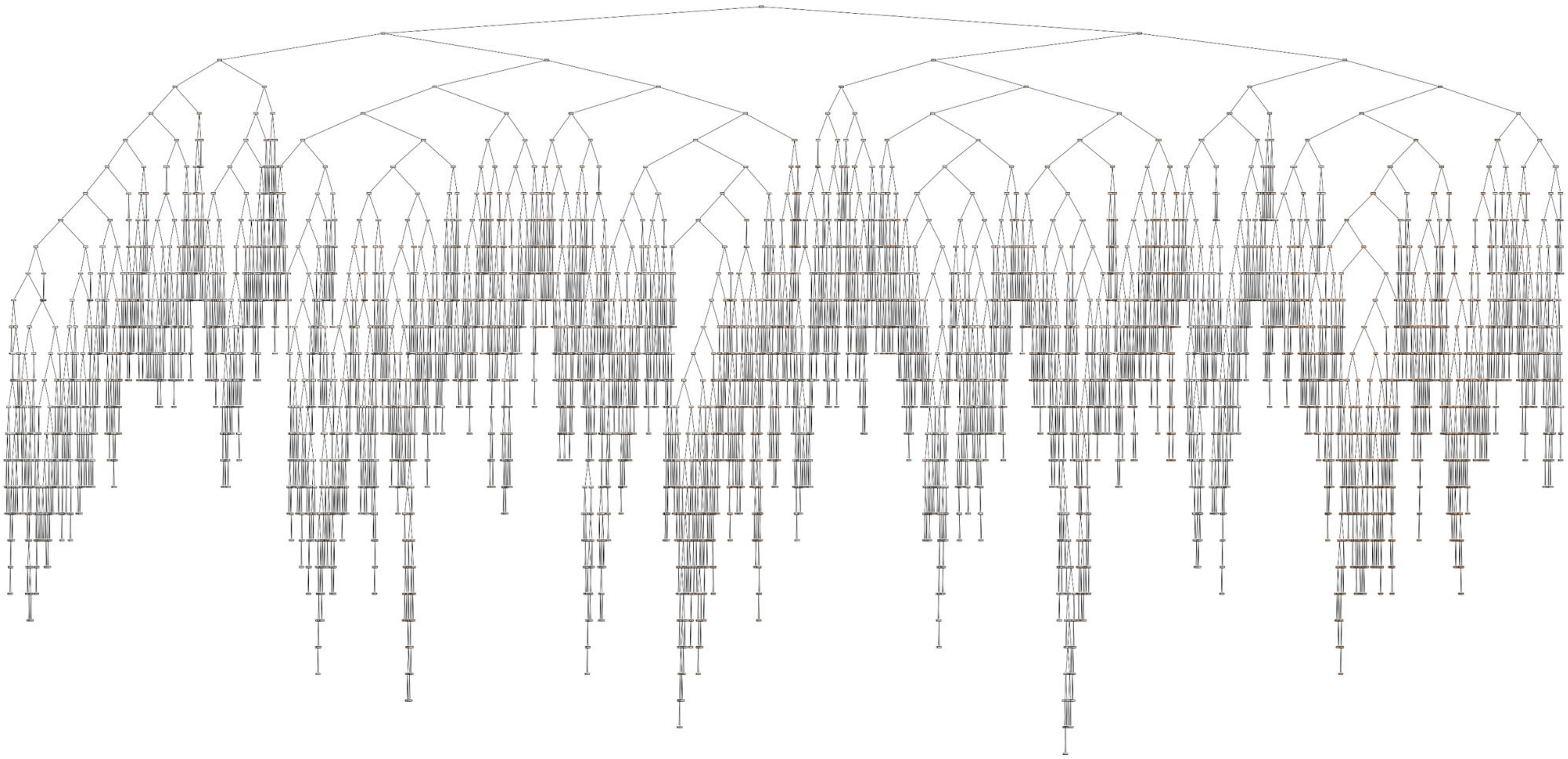

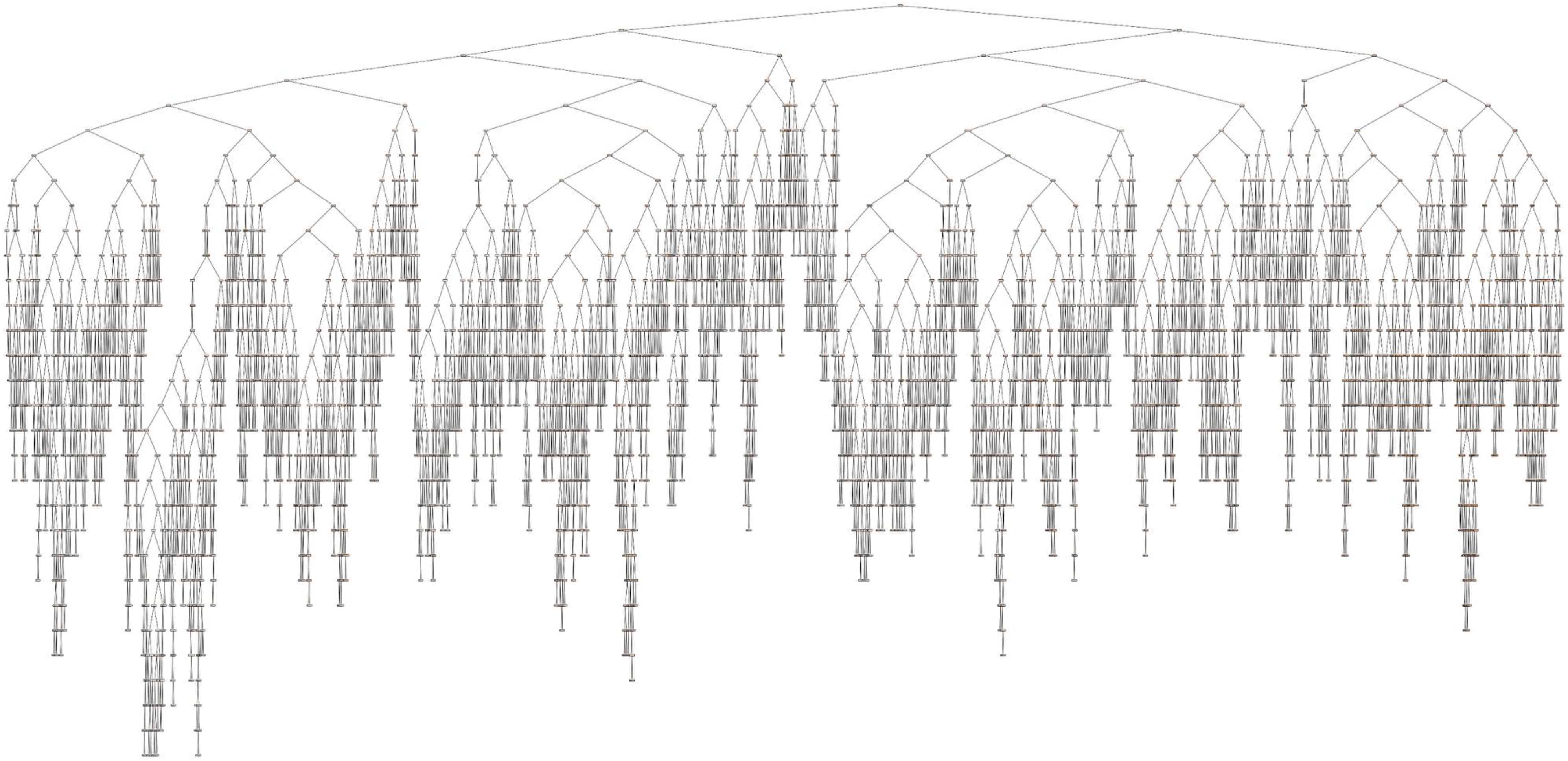

Supplement: Supplementary file 1 [file ao5c08795_si_001.pdf]
